# Inhibition of Rumen Methanogenesis and Ruminant Productivity: A Meta-Analysis

**DOI:** 10.3389/fvets.2018.00113

**Published:** 2018-06-19

**Authors:** Emilio M. Ungerfeld

**Affiliations:** Coordinación de Sistemas Ganaderos, Instituto de Investigaciones Agropecuarias INIA Carillanca, Temuco, Chile

**Keywords:** rumen, methane, methanogenesis, energy, inhibition, ruminants, productivity, meta-analysis

## Abstract

Methane (CH_4_) formed in the rumen and released to the atmosphere constitutes an energy inefficiency to ruminant production. Redirecting energy in CH_4_ to fermentation products with a nutritional value to the host animal could increase ruminant productivity and stimulate the adoption of CH_4_-suppressing strategies. The hypothesis of this research was that inhibiting CH_4_ formation in the rumen is associated with greater ruminant productivity. The primary objective of this meta-analysis was to evaluate how inhibiting rumen methanogenesis relates with the efficiencies of milk production and growth and fattening. A systematic review of peer-reviewed studies in which rumen methanogenesis was inhibited with chemical compounds was conducted. Experiments were clustered based on research center, year of publication, experimental design, feeding regime, type of animal, production response, inhibitor of CH_4_ production, and method of CH_4_ measurement. Response variables were regressed against the random experiment effect nested in its cluster, the random effect of the cluster, the linear and quadratic effects of CH_4_ production, and the random interaction between CH_4_ production and the experiment nested in the cluster. When applicable, responses were adjusted by intake of different nutrients included as regressors. Inhibiting rumen methanogenesis tended to associate positively with milk production efficiency, although the relationship was influenced by individual experiments. Likewise, a positive relationship between methanogenesis inhibition and growth and fattening efficiency depended on the inclusion and weighting of individual experiments. Inhibiting rumen methanogenesis negatively associated with dry matter intake. Interpretation of the effects of inhibiting methanogenesis on productivity is limited by the availability of experiments simultaneously reporting energy losses in feces, H_2_, urine and heat production, as well as net energy partition. It is concluded that inhibiting rumen methanogenesis has not consistently translated into greater animal productivity, and more animal performance experiments are necessary to better characterize the relationships between animal productivity and methanogenesis inhibition in the rumen. A more complete understanding of changes in the flows of nutrients caused by inhibiting rumen methanogenesis and their effect on intake also seems necessary to effectively re-channel energy gained from CH_4_ suppression toward consistent gains in productivity.

## Introduction

Ruminants are important to humans in converting non-usable forages to products such as meat, milk, wool and traction. The mixed rumen microbiota has the ability to digest plant fiber unavailable for humans and produce fermentation products and microbial biomass that the host animal absorbs and converts to products useful for humans. Methane (CH_4_) is the main sink of metabolic hydrogen ([H]) in rumen fermentation. Metabolism of carbohydrates by the fermentative microbiota of bacteria, protozoa and fungi reduces co-factors, which are re-oxidized mostly by transferring electrons to protons. Dihydrogen (H_2_) so formed is transferred to methanogenic Archaea, which utilize it to reduce carbon dioxide (CO_2_) to CH_4_ (1).

In recent years, there has been considerable research efforts to control the formation of CH_4_ in the rumen, with the objective of ameliorating CH_4_ emissions from domestic ruminants. Agriculture accounts for between 10 and 12% of global emissions of greenhouse gases expressed as CO_2_-equivalents, with the largest contributor being enteric CH_4_ (2). Because CH_4_ has a global warming potential 28- to 34-fold greater than CO_2_, decreases in anthropogenic CH_4_ emissions are a strategic target for ameliorating climate change (3).

In addition, CH_4_ emissions represent an energy loss to the animal of between 2 and 12% of gross energy intake (GEI) of ruminants (4). Historically, research on the inhibition of rumen methanogenesis started driven by scientists identifying the formation of CH_4_ in the rumen as an energy inefficiency: “It is argued that if the methane production could be inhibited specifically, rumen fermentation might change toward greater efficiency. Methane is produced from carbon dioxide and metabolic hydrogen and the manipulation could be effective if the hydrogen saved from methanogenesis could be used in formation of products that might subsequently be used by the host animal” (5). Redirecting [H] from CH_4_ toward propionate has been proposed as a means to increase the amount of ME available to the animal (6). Energy lost as CH_4_ is an inefficiency in the conversion of digestible energy (DE) to metabolizable energy (ME), because CH_4_ is formed from organic matter (OM) digested and fermented in the rumen and hindgut (7).

Unless in the future CH_4_ mitigation strategies become mandatory or stimulated by government subsidies, it seems unlikely that they will be widely implemented by producers if their adoption is not profitable (8). Thus, if energy lost as CH_4_ could be capitalized by incorporating it into products that the host animal can absorb and use, producers would more likely adopt strategies to ameliorate CH_4_ emissions. Gains in productivity are therefore considered critical, and an opportunity, for the design of CH_4_ mitigation strategies that are at the same time economically beneficial to producers.

Meta-analyses relating the dietary content of fats and oils (9–11), monensin (12), nitrate (13), and 3-nitroxypropanol (14) to CH_4_ emissions and animal performance, digestion and metabolism, have generated useful applied knowledge about the effectiveness of those ingredients and additives for CH_4_ mitigation. Apart from dose response analyses, the overall biological response of animal productivity to the methanogenesis inhibition intervention has to the author's knowledge yet to be studied. Patra (15) meta-analyzed the effects of inhibiting CH_4_ production with phytochemicals on digestion and fermentation. The present analysis evaluates the effects on ruminant productivity and energy partition of decreasing CH_4_ production in the rumen.

Various strategies are being investigated to control CH_4_ production by ruminants. Some CH_4_-abattement strategies, such as dietary changes or selection of more efficient animals, may also impact productivity through means unrelated to methanogenesis inhibition; experiments using these CH_4_-abattement strategies would thus not be suitable to examine the effects of inhibiting rumen methanogenesis on animal productivity in isolation. In order to understand the effects of decreasing CH_4_ production in the rumen on ruminant productivity unmasked by other factors, the present analysis focuses only on experiments in which methanogenesis was specifically targeted using chemical inhibitors. It was hypothesized herein that inhibiting CH_4_ production with chemical compounds enhances animal productivity through improving energy use efficiency. The objectives of this meta-analysis were: (1) To examine using meta-analysis of published results if inhibiting rumen methanogenesis with specific chemical compounds has associated with improvements in the efficiencies of milk production and growth and fattening; (2) To interpret the relationships between rumen methanogenesis inhibition and the efficiencies of milk production and growth and fattening by examining how inhibiting rumen methanogenesis has associated with energy losses in digestion and metabolism.

## Methods

### Search criteria

Peer-reviewed publications published in English reporting original research on the inhibition of rumen methanogenesis *in vivo* using chemical compounds were searched in PubMed (https://www.ncbi.nlm.nih.gov/pubmed/), Web of Science (https://apps.webofknowledge.com/WOS_GeneralSearch_input.do?product=WOS&search_mode=GeneralSearch&SID=5C93Gu6M6z6HUIaDDxS&preferencesSaved=) and Agricola (https://agricola.nal.usda.gov/). Articles were searched in the databases based on the following keywords present in their title or abstract or keywords using the following Boolean operation: (rumen OR ruminant OR ruminants OR dairy OR beef OR sheep OR goats OR buffaloes) AND methane AND inhibition. Also, articles on the inhibition of rumen methanogenesis were obtained from the MitiGate database (2) and the author's personal files. A total of 89, 280, 121, and 333 records were retrieved from PubMed, Web of Science, Agricola and MitiGate, respectively, many of which were present in more than one databases.

### Study eligibility criteria

Most of the records retrieved referred to *in vitro* experiments and were not used in the analysis. Of the *in vivo* studies, only those ones reporting experiments in which rumen methanogenesis was inhibited through the use of specific chemical inhibitors were used to study how inhibiting rumen methanogenesis affected ruminant productivity. Use of chemical additives with known composition and included in relatively small amounts in the diet is thought to be the most likely CH_4_ amelioration intervention which could affect animal productivity solely through inhibiting methanogenesis, although it is acknowledged that some antimethanogenic chemicals can be toxic to microorganisms other than methanogens and might therefore cause other effects. Other approaches to ameliorate CH_4_ emissions are less specific and might affect animal productivity through means unrelated to methanogenesis inhibition: dietary manipulation (augmented supply of nutrients or improved nutrient balance), ionophores [improved N utilization efficiency (16)], essential oils [decreased protein and starch degradation (17)], lipid supplementation [enhanced energy supply vs. lesser intake, fiber digestibility and inhibition of *de novo* milk fatty acids synthesis (10, 11)], defaunation [increased microbial protein production; (18)], tannins and saponins [improved supply of protein digested in the small intestine; (19)], alternative [H] sinks [provision of extra fermentable energy; (20)], and enzyme supplementation [improved fiber digestibility; (11)].

Selecting animals with better feed conversion efficiency has resulted in animals with lower CH_4_ production (21). The results from the study by Fitzsimons et al. (21) were not included in the present meta-analysis however, because the approach was reversal in the sense that less CH_4_ production was a consequence of greater productivity, rather than enhanced productivity a consequence of methanogenesis inhibition.

Immunization against methanogens is being studied as a potential anti-methanogenic strategy (22), but results on the effects of immunization against methanogens on milk production or bodymass change, i.e., animal productivity were not found in the present literature search.

Experiments in which nitrate replaced urea as a source of non-protein N were included in the analysis, with the understanding that neither urea or nitrate would contribute dietary gross energy (GE) available to the ruminant, or nutrients other than N. In experiments in which nitrate was used as an inhibitor of CH_4_ production, the diet composition was carefully checked to ensure that either analyzed organic matter (OM) or GE did not differ by more than 2%, or that, if analyzed dietary OM or GE content were not provided, that nitrate did not replace dietary true protein.

### Database

After discarding studies examining strategies to ameliorate CH_4_ production by ruminants other than chemical inhibitors, a total of 75 studies including 96 experiments in which rumen methanogenesis *in vivo* was inhibited with chemical additives was obtained. Of these, 44 studies (23–66) with 54 experiments including a total of 163 treatments were used as the final database for the meta-regressions (Table S1), and 42 experiments belonging to 31 studies were discarded because of different reasons detailed in Table S2.

Response variables analyzed were:

1. Milk production efficiency (10 experiments, 26 treatment means), defined as energy-corrected milk (ECM) production adjusted by dry matter intake (DMI). When not provided, ECM production was calculated from milk production (kg) and milk content of fat, protein and lactose (67);
2. Growth and fattening efficiency (13 experiments, 38 treatment means) defined as bodymass gain (BMG) adjusted by DMI;
3. Digestion and metabolism variables: DMI (*ad libitum* intake experiments only). Feces output of DM (DMf), OM (OMf), N (Nf), and NDF (NDFf) adjusted by intake of DM, OM, N, and NDF, respectively. Energy losses in feces (EF), gases (EG; CH_4_ + H_2_), urine (UE), and heat (HP), all adjusted by GEI. Rumen pH, total rumen volatile fatty acids (VFA) concentration, individual VFA molar percentages, rumen ammonium concentration, and total bacteria, protozoa and methanogens *16S rRNA, 18S rRNA*, and *16S rRNA* or *mcrA* gene copies, respectively (log_10_/mL rumen contents)

### Clusters

In meta-analysis, estimates of effect sizes belonging to different experiments are not independent due to the fact that experiments differ to different degrees in various aspects, such as research methods employed, research group, animals, location etc. (68). The approach taken herein to account for lack of independence was to model dependence by introducing clusters of experiments in the meta-regressions (69).

Experiments were grouped for each response variable according to clustering variables related to experimental variables detailed in Table S3: research center, year of publication, experimental design, feeding regime, type of animal, production response, inhibitor of CH_4_ production and method of CH_4_ measurement. Hierarchical clusters were built for each response variable using the Ward method with standardized data with JMP 13.2.1.

### Regressions

Response variables were meta-regressed against the random effect of the experiment (70) nested in the cluster, the linear and quadratic effects of CH_4_ production, and the random linear interaction between the experiment and CH_4_ production nested in the cluster. The general model was:

(1) $$\begin{array}{rcl} Y_{ijk} & = & \mu +exp\left[cluster\right]{\left(random\right)}_{\text{ij}}+cluster{\left(random\right)}_{\text{j}}\\ +\beta_{1}CH_{4\text{ijk}}+\beta_{2}CH_{4\text{ijk}}^{2}+ECH_{4\text{ijk}}+res_{ijk} \end{array}$$

Where *Y*_*ijk*_ is the treatment mean k of a response variable of interest of the experiment i nested in cluster j, μ is the overall intercept, *exp*(random, cluster)_ij_ is the random effect of experiment i nested in cluster j, cluster(random)_j_ is the random effect of the cluster j, β_1_ and β_2_ are the overall linear and quadratic regression coefficients of CH_4_ production on *Y*, respectively, CH_4ijk_ and ${\text{CH}}_{4}^{2}$_ijk_ are the treatment mean and treatment mean squared of CH_4_ production of treatment k of experiment i nested in cluster j, respectively, E is the random effect of the experiment i nested in cluster j on β_1_, and *res*_*ijk*_ is the residual of treatment mean k of experiment i nested in cluster j, assumed to be normally distributed with mean equal to 0 and variance σ.

When production of gases (CH_4_ or H_2_) was reported as grams per day, it was converted to liters per day using the molar mass of each gas and the General Law of Gases assuming an absolute temperature of 298 K.

Milk and growth and fattening production efficiencies can be evaluated as the quotient of ECM production or BMG, respectively, to DMI. However, the use of ratios as response variables can be problematic because of correlations between the regressors and the numerator or denominator variable in the ratio (71), with, in the present analysis, CH_4_ production being largely driven by DMI (72). Therefore, the approach taken for modeling milk and growth and fattening production efficiencies was to regress daily ECM production and BMG against CH_4_ production adjusted for DMI, as follows:

(2) $$\begin{array}{rcl} Y_{ijk} & = & \mu +exp\left[cluster\right]{\left(random\right)}_{\text{ij}}+cluster{\left(random\right)}_{\text{j}}\\ +\beta_{0}DMI_{\text{ijk}}+\beta_{1}CH_{4ijk}+\beta_{2}CH_{4\text{ijk}}^{2}+ECH_{4\text{ijk}}+res_{ijk} \end{array}$$

Where *Y*_*ikj*_ is the treatment mean k of ECM or BMG of the experiment i nested in cluster j, β_0_ is the overall linear regression coefficient of DMI, DMI_ijk_ is the DMI of treatment k in experiment i of cluster j, with the rest of the variables and parameters in model (2) defined as in model (1).

Similarly, the effects of inhibiting rumen methanogenesis on digestibility of DM, OM, N, and NDF were studied by regressing the fecal daily outputs of dry matter (DMf), organic matter (OMf), nitrogen (Nf), or NDF (NDFf) adjusted by their daily intakes against CH_4_ production as follows:

(3) $$\begin{array}{rcl} Y_{ijk} & = & \mu +exp\left[cluster\right]{\left(random\right)}_{\text{ij}}+cluster{\left(random\right)}_{\text{j}}+\beta_{0}X_{\text{ijk}}\\ +\beta_{1}CH_{4\text{ijk}}+\beta_{2}CH_{4\text{ijk}}^{2}+ECH_{4\text{ijk}}+res_{ijk} \end{array}$$

Where *Y*_*ikj*_ is the treatment mean k of DMf, OMf, Nf, or NDFf in experiment i nested in cluster j, β_0_ is the overall regression coefficient of DMI, organic matter intake (OMI), nitrogen intake (NI), or NDF intake (NDFI), respectively, *X*_ijk_ is the intake of DM, OM, N, or NDF of treatment k in experiment i of cluster j, respectively, with the rest of the variables and parameters in model (3) defined as in models (1) and (2).

Likewise, responses of energy losses in feces (EF), total gases (CH_4_ + H_2_, EG), urine (UE), and heat (HP) were adjusted by GEI included as a regressor:

(4) $$\begin{array}{rcl} Y_{ijk} & = & \mu +exp\left[cluster\right]{\left(random\right)}_{\text{ij}}+cluster{\left(random\right)}_{\text{j}}+\beta_{0}GEI_{\text{ijk}}\\ +\beta_{1}CH_{4\text{ijk}}+\beta_{2}CH_{4\text{ijk}}^{2}+ECH_{4\text{ijk}}+res_{ijk} \end{array}$$

Where *Y*_*ijk*_ corresponds to EF, EG, UE or HP of treatment k in experiment i nested in cluster j, β_0_ is the overall regression coefficient of GEI, GEI_ijk_ is the mean of GEI of treatment k in experiment i nested in cluster j, with the rest of the variables and parameters in model (4) defined as in models (1), (2), and (3). Energy in CH_4_, feces, total gases, urine and heat were expressed in MJ/d. Heats of combustion were obtained from Domalski (73) and McAllister et al. (74).

Fixed effects with *P* < 0.05 were considered significant, and those with 0.05 ≤ *P* ≤ 0.10 were considered tendencies. Random interactions experiment by CH_4_ nested in the cluster with Wald *P* < 0.05 were considered significant, and those with 0.05 ≤ *P* ≤ 0.10 were considered tendencies. Quadratic and interaction effects with *P* > 0.10 were eliminated and the reduced model re-fitted.

In meta-regressions, it is recommended to weight treatment means by the reciprocal of their standard errors (SEM) normalized to unity (75). However, difficulties encountered for conducting this procedure were: (1) Treatment means of ECM were calculated as compound variables from reported milk production and composition, and their SEM is not calculable; (2) SEM are not provided in some of the older studies (24, 27, 29). An alternative weighting scheme, such as the number of animals in the experiment, could not be used because the present analysis included experiments with both randomized plots and blocks, and Latin Squares and cross over designs, the latter obviously including fewer animals. Therefore, regressions were conducted in first instance with unweighted treatment means. As a precaution against the possibility of experiments with few animals or high experimental error having excessive influence in the results, special attention was paid to experiments not reporting SEM when examining the results for the presence of outliers and influential observations (see Analysis of outliers and influential treatment means).

As a second precaution against some experiments having excessive influence on the regressions, SEM of ECM were estimated as linear combinations of the SEM of milk production and composition, acknowledging that this proxy is inaccurate. Subsequently, regressions of ECM and BMG against CH_4_ production were re-run as above described but with treatment means weighted by the reciprocal of their estimated SEM normalized to unity, and compared to regressions with unweighted treatment means conducted on the same sub-set of experiments.

### Analysis of experimental co-variables

The potential influence of various experimental co-variables on the response of ECM, BMG and DMI to methanogenesis inhibition was examined by replacing in the regressions the experiment and cluster effects by different co-variables related to the experiment fitted separately, as follows:

(5) $$\begin{array}{r} Y_{k}=\mu +\beta_{0}V_{\text{k}}+\beta_{1}CH_{4\text{k}}+\beta_{2}Z_{\text{k}}+\beta_{3}CH_{4\text{k}}\times Z_{\text{k}}+res_{k} \end{array}$$

Where *Y*_k_ is the k^th^ treatment mean of the response variable across the entire database, μ is the overall intercept, β_0_ is the regression coefficient of the intake of the fraction corresponding to the response variable (i.e., intake of DM, OM, N, NDF, or energy), *V*_k_ is the DM, OM, N, NDF or energy intake of treatment k across the entire database, β_1_ and β_2_ are the linear and quadratic regression coefficients of CH_4_ production on *Y*, respectively, CH_4k_ and ${\text{CH}}_{4}^{2}$_k_ are the treatment mean and treatment mean squared of the overall k level of CH_4_ production, β_3_ is the regression coefficient of experimental co-variable *Z, Z*_k_ is the treatment mean of the overall k level of *Z*, β_4_ is the regression coefficient of the interaction between CH_4k_ and *Z*_k_, and *res*_*k*_ is the residual of the overall k level of CH_4_ production and *Z*, assumed to be normally distributed with mean equal to 0 and variance σ.

The interaction effect between methanogenesis inhibition and classification type of co-variables on each response variable was studied only for co-variables in which each level of the co-variable was represented in at least two experiments.

Co-variables analyzed were: type of animal (dairy cows, goats, growing heifers, steers, or sheep), feeding regime (restricted or *ad libitum*; except for DMI), stage of lactation at the beginning of the experiment (for ECM only), type of experimental design (fixed assignment of animals to treatments or treatment switch), type of response (maintenance, milk production or growth and fattening), type of methanogenesis inhibitor, and content of dietary concentrate, N and NDF (DM basis).

### Analysis of outliers and influential treatment means

Homoscedasticity was examined through residual against predicted plots. The assumption of residuals normality was examined through residual normality plots. Outliers were identified as those treatment means whose absolute value studentized residuals were greater than *t*_N−p−1, 0.95_, with p being the number of parameters and *N* the number of treatment means. Influential treatment means were identified as those with a leverage value larger than 2p/N (76). Experiments containing outliers and/or influential treatment means were deleted one at a time and regressions re-fitted in their absence. If the conclusions of the analysis changed after the deletion of experiments containing outliers and/or influential treatment means (significant effects became non-significant or vice versa, or the direction of the response changed), the results are presented and discussed both with and without the experiments containing the outliers and/or influential observations.

JMP® 13.2.1 (SAS Institute, Cary, NC, USA) was used for all statistical analyses.

## Results

### Descriptive statistics, clusters of experiments, and methanogenesis inhibition

A summary of statistics of regressors and response variables is presented in Table 1. The clustering details for the main response variables are presented in Table S4.

**Table 1:** Summary of statistics of regressors and response variables.

**Variable**	***N***	**Number of experiments**	**Mean**	***SD*^b^**	**Range**
N (%DM)^a^	137	44	2.33	0.54	0.8–3.51
Concentrate (%DM)	141	46	43.6	24.0	0–90.5
NDF (%DM)	109	33	40.5	11.4	19.2–76.5
CH_4_ (L/d)	163	54	168	178	0–744
CH_4_/DMI (L/kg MS)	151	49	23.3	10.1	0–44.8
DMI (kg/d)^c^	83	25	10.8	6.89	0.99–28
ECM (kg/d)	26	10	27.6	10.8	1.17–46.2
ECM/DMI (kg/kg DM)	26	10	1.41	0.17	1.14–1.81
BMG (kg/d)	38	13	0.75	0.44	0.022–1.55
BMG/DMI (kg/kg DM)	38	13	0.11	0.044	0.025–0.17
DMD (%)	46	17	68.6	4.48	56.8–76.4
DMf (kg/d)	44	16	6.91	6.09	0.46–19.8
OMD (%)	37	13	71.1	3.98	62–79.2
OMf (kg/d)	30	10	9.11	5.37	0.44–18.5
ND (%)	38	14	69.1	7.28	53.4–82.9
Nf (kg/d)	34	12	0.20	0.17	0.019–0.49
NDFD (%)	34	12	53.8	8.30	30.7–64.9
NDFf (kg/d)	34	12	2.50	1.16	0.18–4.84
EF (MJ/100 MJ GEI)	44	17	29.5	4.79	14.1–35
EF (MJ/d)	31	12	27.0	29.0	1.56–89.9
H_2_ (L/d)	54	15	37.6	78.3	0–193
H_2_ (MJ/d)	38	9	0.41	1.04	0–2.26
EG (MJ/100 MJ GEI)	18	6	5.28	1.57	2.7–8.4
EG (MJ/d)	38	9	9.67	7.46	0.34–27.1
UE (MJ/100 MJ GEI)	36	14	3.70	1.33	1.20–6.4
UE (MJ/d)	29	11	2.89	2.69	0.19–8.7
HP (MJ/100 MJ GEI)	21	8	42.6	9.76	27.7–61.8
HP (MJ/d)	21	8	53.7	48.1	5.49–138
Rumen pH	50	17	6.50	0.32	5.5–7.33
Rumen total VFA (mM)	86	30	89.8	24.9	47.5–161
Acetate (mM)	86	30	55.3	15.4	31–99.2
Propionate (mM)	86	30	18.2	6.42	6.49–38.5
Butyrate (mM)	86	30	10.9	4.31	4.73–23.5
Isobutyrate (mM)	54	18	1.69	1.52	0.48–7.46
Valerate (mM)	63	22	2.01	1.22	0.36–6.04
Isovalerate (mM)	59	20	2.04	1.12	0.12–4.76
Acetate/propionate (mM/mM)	67	24	3.32	0.90	1.95–6.1
${\text{NH}}_{4}^{+}$ (mM)	65	22	9.65	7.66	1.64–30
Bacteria (log_10_ *16S rRNA* gene copies/g rumen contents)	27	10	10.4	1.55	7.03–12.5
Protozoa (log_10_ *18S rRNA* gene copies/g rumen contents)	25	9	6.44	2.76	1.46–12.0
Methanogens [log_10_ (*16S rRNA* + *mcrA*) gene copies/g rumen contents]	27	10	7.92	2.28	2.47–11.9

a *BMG, bodymass gain; CH_4_, methane; DM, dry matter; DMD, dry matter digestibility; DMf, dry matter output in feces; DMI, dry matter intake; ECM, energy-corrected milk; EF, energy output in feces; EG, energy output in gases; GEI, gross energy intake; H_2_, dihydrogen; HP, heat production; N, nitrogen; ND, nitrogen digestibility; NDF, Neutral detergent fiber; NDFD, neutral detergent fiber digestibility; Nf, nitrogen output in feces; ${\text{NH}}_{4}^{+}$, ammonium; OMD, organic matter digestibility; OMf, organic matter output in feces; UE, urine energy; VFA, volatile fatty acids*.

b *Standard deviation*.

c *Only experiments with ad libitum feeding considered*.

Conceptually, the degree of methanogenesis inhibition is the reverse of CH_4_ production i.e., the greater methanogenesis inhibition the lesser CH_4_ production. Therefore and because of the main objective of this analysis was to understand the how the intervention of inhibiting rumen methanogenesis associates with animal productivity, results are presented and discussed in terms of the relationships between methanogenesis inhibition and the different response variables.

### Milk production efficiency

**Figure 1:**
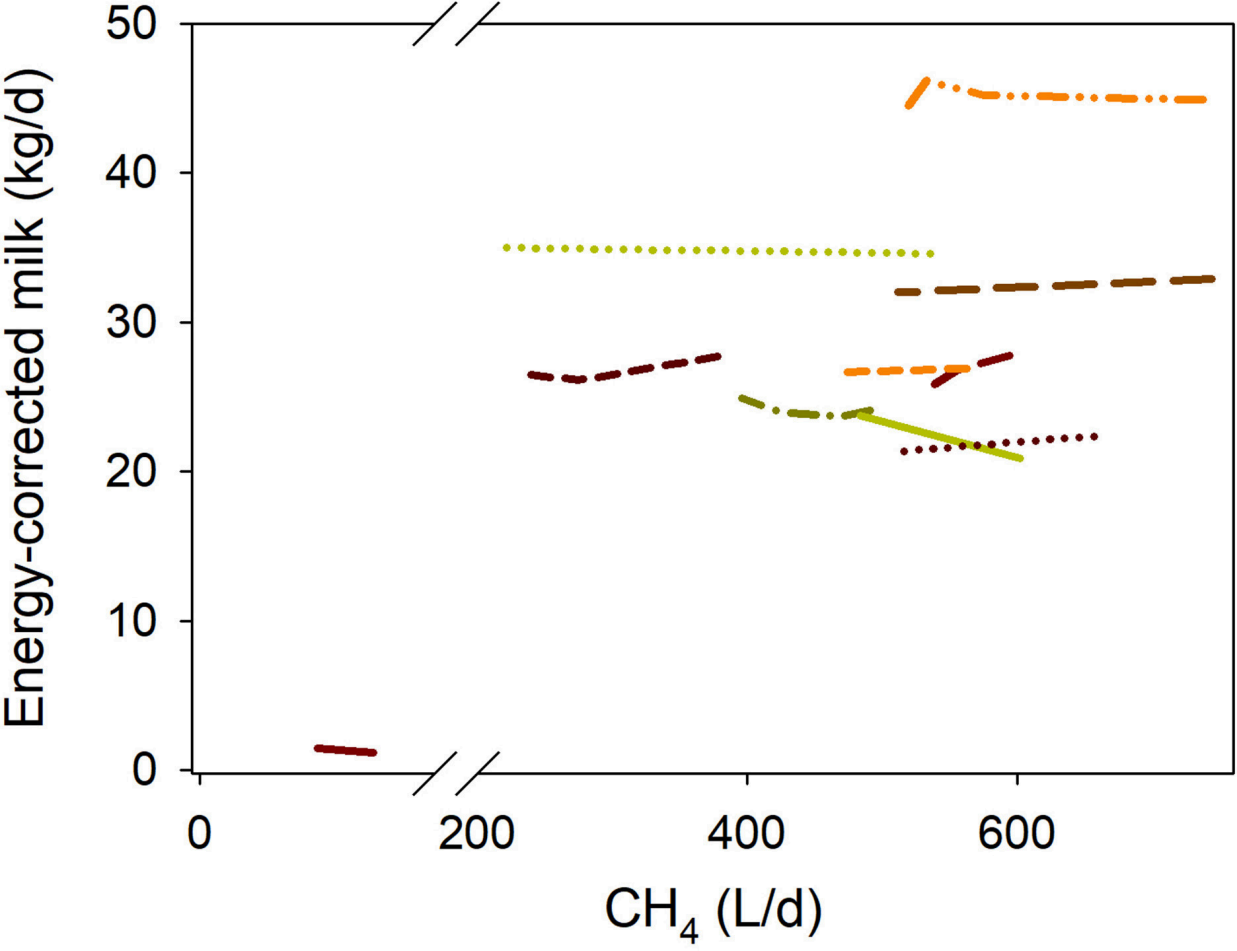
Response of energy-corrected milk (ECM, kg/d) production adjusted by dry mater intake (DMI, kg/d)] to methanogenesis inhibition, including the random effect of the experiment nested in the cluster, and the random effect of the cluster. Each separate line corresponds to a different experiment: (i) Unweighted treatment means: ECM = −0.46 (±3.22; *P* = 0.89) + 1.50 (±0.18; *P* < 0.001) DMI – 0.0014 (±0.0023; *F* = 0.34, *P* = 0.57) CH_4_; *N* = 26, 10 experiments; (ii) Weighted treatment means: ECM = −1.17 (±2.61; *P* = 0.67) + 1.67 (±0.16; *P* < 0.001) DMI – 0.0067 (±0.0037; *F* = 3.33, *P* = 0.084) CH_4_; *N* = 26, 10 experiments.

There was no relationship between inhibiting methanogenesis and DMI-adjusted ECM production (*P* = 0.57; Figure 1 i). If the regression was weighted by the estimated reciprocal of the treatment means SEM normalized to unity, there was a tendency (*P* = 0.084) toward a positive association between DMI-adjusted ECM production and methanogenesis inhibition (Figure 1 ii). A sensitivity analysis found that this tendency became a significant positive association (*P* = 0.007; not shown) if eliminating the experiment by van Zijderveld et al. (40), and was not significant (*P* = 0.67; not shown) if excluding the first experiment by Veneman et al. (59).

There were no interactions between methanogenesis inhibition and type of animal (*P* = 0.97), experimental design (*P* = 0.49), feeding regime (*P* = 0.67), dietary N (*P* = 0.86) or NDF (*P* = 0.28), or type of methanogenesis inhibitor (*P* = 0.77) on DMI-adjusted ECM. There was a tendency (*P* = 0.091) toward a positive association between DMI-adjusted ECM and methanogenesis inhibition with greater dietary concentrate (not shown).

### Growth and fattening efficiency

**Figure 2:**
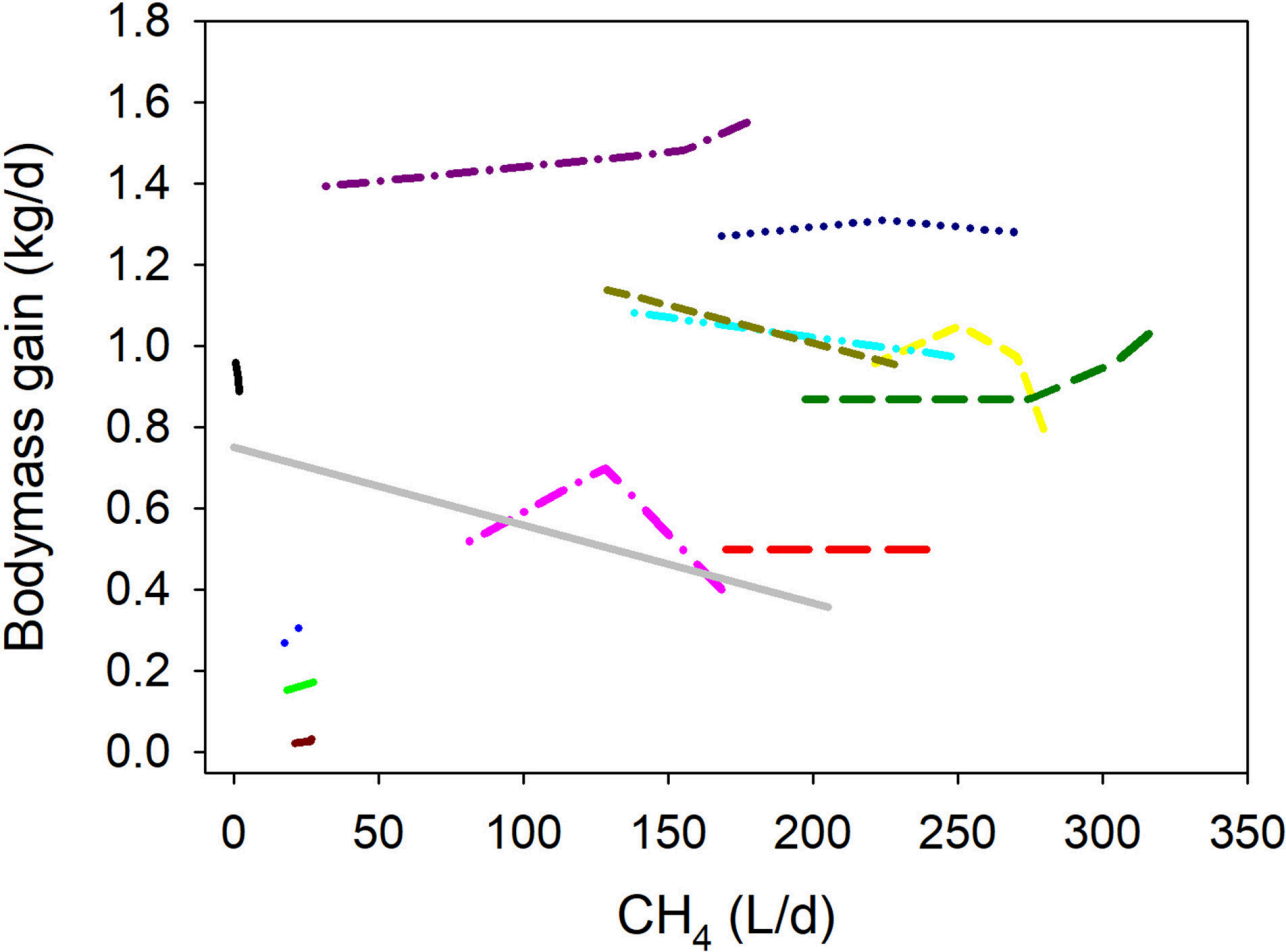
Response of bodymass gain (BMG, kg/d) adjusted by dry matter intake (DMI, kg/d) to methanogenesis inhibition, including the random effect of the experiment nested in the cluster, and the random effect of the cluster. Each separate line corresponds to a different experiment: (i) Unweighted treatment means (all experiments): BMG = 0.026 (±0.14; *P* = 0.86) + 0.13 (±0.021; *P* < 0.001) DMI – 0.0010 (±0.00032; *F* = 10.8, *P* = 0.003) CH_4_; *N* = 38, 13 experiments; (ii) Weighted treatment means: BMG = 0.036 (±0.15; *P* = 0.81) + 0.11 (±0.023; *P* < 0.001) DMI – 0.00038 (±0.00034; *F* = 1.29, *P* = 0.27) CH_4_; *N* = 32, 11 experiments; iii) Unweighted treatment means: BMG = 0.0071 (±0.16; *P* = 0.96) + 0.13 (±0.023; *P* < 0.001) DMI – 0.00096 (±0.00036; *F* = 7.18, *P* = 0.013) CH_4_; *N* = 32, 11 experiments.

Inhibiting CH_4_ production associated positively with DMI-adjusted BMG (*P* = 0.003; Figure 2 i). If the regression was weighted by the reciprocal of the treatment means SEM normalized to unity, there was no relationship between CH_4_ production and DMI-adjusted BMG (*P* = 0.27; Figure 2 ii). If the regression was conducted in the same sub-set of experiments for which SEM were available [i.e., excluding the experiments by Davies et al. (29) and Tomkins et al. (36), but without weighting the treatment means, inhibiting CH_4_ production associated positively with DMI-adjusted BMG (*P* = 0.013; Figure 2 iii); in the latter case, a sensitivity analysis found no relationship (*P* = 0.25) between CH_4_ production and DMI-adjusted BMG if the experiment by McCrabb et al. (31) was excluded from the analysis.

There were no interactions between methanogenesis inhibition and type of animal (*P* = 0.93), experimental design (*P* = 0.24), feeding regime (*P* = 0.31), dietary concentrate (*P* = 0.84), dietary N (*P* = 0.69), or NDF (*P* = 0.97), or type of methanogenesis inhibitor (*P* > 0.75) on DMI-adjusted BMG (not shown).

### Dry matter intake and digestibility

**Figure 3:**
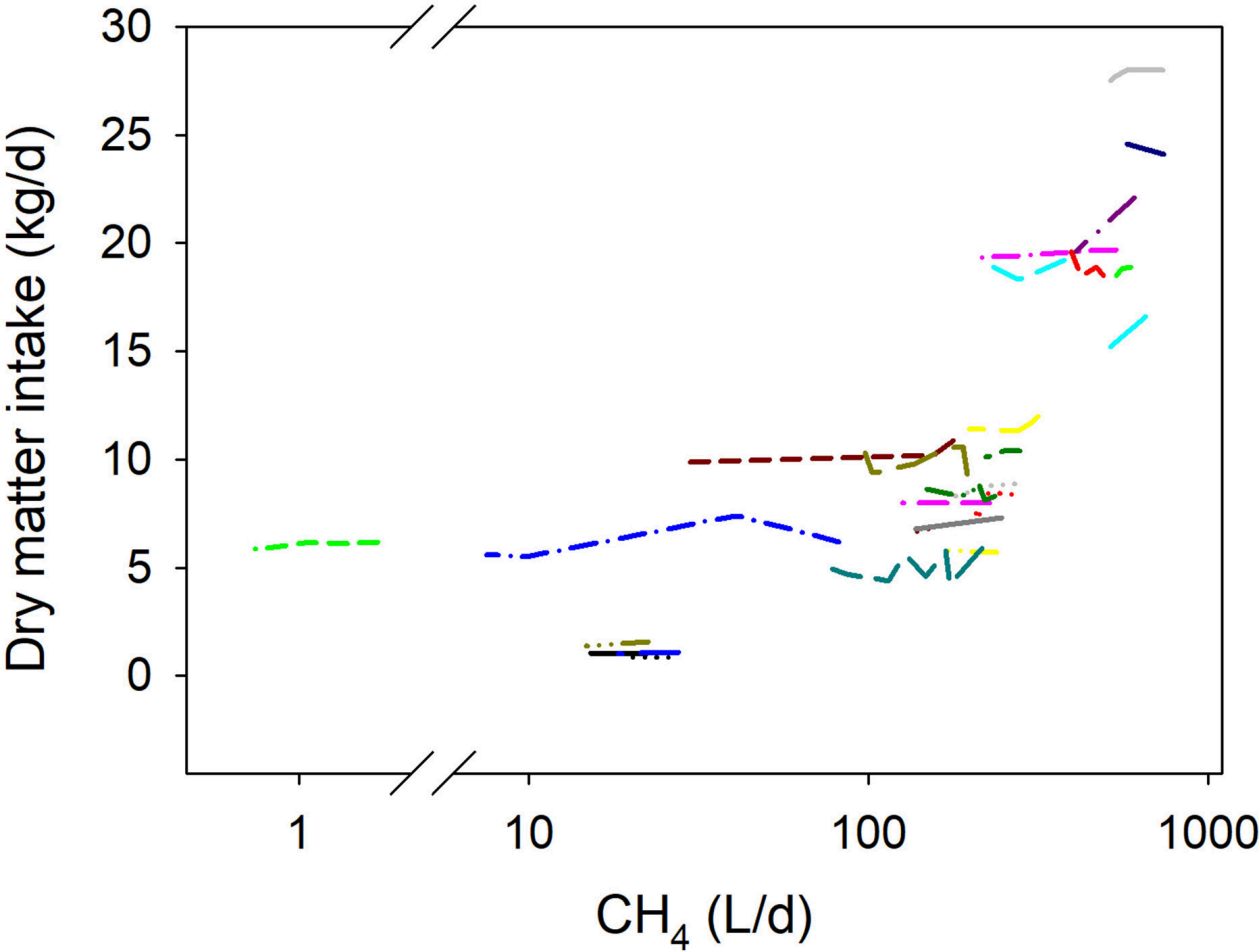
Response of dry matter intake (DMI, kg/d) to methanogenesis inhibition, including the random effect of the experiment nested in the cluster and the random effect of the cluster. Each separate line corresponds to a different experiment: DMI = 9.99 (±2.08; *P* = 0.001) + 0.0036 (±0.00099; *F* = 13.4, *P* < 0.001) CH_4_; *N* = 83, 25 experiments.

Inhibiting methanogenesis associated with decreased DMI (*P* < 0.001; Figure 3). The predicted DMI at a theoretical 100% methanogenesis inhibition was 10% lower than the mean of control treatments (not shown). There were no interactions between methanogenesis inhibition and type of animal (*P* = 0.99), production response (*P* = 0.25), and dietary content of concentrate (*P* = 0.26), or N (*P* = 0.50). There was a tendency (*P* = 0.051) toward a more negative relationship between DMI and methanogenesis inhibition in experiments with a randomized design than in those in which animals were switched between diets, and with lesser content of dietary NDF (*P* < 0.001) (not shown).

There were no relationship between methanogenesis inhibition and DMI-adjusted DMf (*P* = 0.72; Table 2). A sensitivity analysis found a tendency (*P* = 0.086) toward greater DMI-adjusted DMf with methanogenesis inhibition if the experiment by Reynolds et al. (52) was removed (not shown). There were no relationships between inhibiting CH_4_ production and OMI-adjusted OMf (*P* = 0.84), NI-adjusted Nf (*P* = 0.58), or NDFI-adjusted NDFf (*P* = 0.83; Table 2).

**Table 2:** Effects of inhibiting methanogenesis on digestibility of different fractions.

**Equation^a^**	***N***	**Number of experiments**
DMf^b^ (kg/d) = 0.11 (±0.18; *P* = 0.58) + 0.68 (±0.018; *P* < 0.001) DMI – 0.00033 (±0.00090; *F* = 0.13, *P* = 0.72) CH_4_	44	16
OMf (kg/d) = 0.35 (±0.56; *P* = 0.68) + 0.68 (±0.037; *P* = 0.016) OMI – 0.00020 (±0.00095; *F* = 0.044, *P* = 0.84) CH_4_	30	10
Nf (kg/d) = 0.0018 (±0.0041; *P* = 0.68) + 0.64 (±0.020; *P* < 0.001) NI – 1.29 × 10^−5^ (±2.30 × 10^−5^; *F* = 0.31, *P* = 0.58) CH_4_	34	12
NDFf (kg/d) = – 0.22 (±0.28; *P* = 0.45) + 0.48 (±0.068; *P* < 0.001) NDFI – 9.53 × 10^−5^ (±0.00044; *F* = 0.047, *P* = 0.83) CH_4_	34	12

a *All regression models include the random effect of the experiment nested in the cluster and the random effect of the cluster*.

b *CH_4_, methane (L/d); DMf, dry matter output in feces; DMI, dry matter intake (kg/d); NDFf, neutral detergent output in feces; NDFI, neutral detergent fiber intake (kg/d); Nf, nitrogen output in feces; NI, nitrogen intake (kg/d); OMf, organic matter output in feces; OMI, organic matter intake (kg/d)*.

### Energy losses in feces, gases, urine, and heat

**Figure 4:**
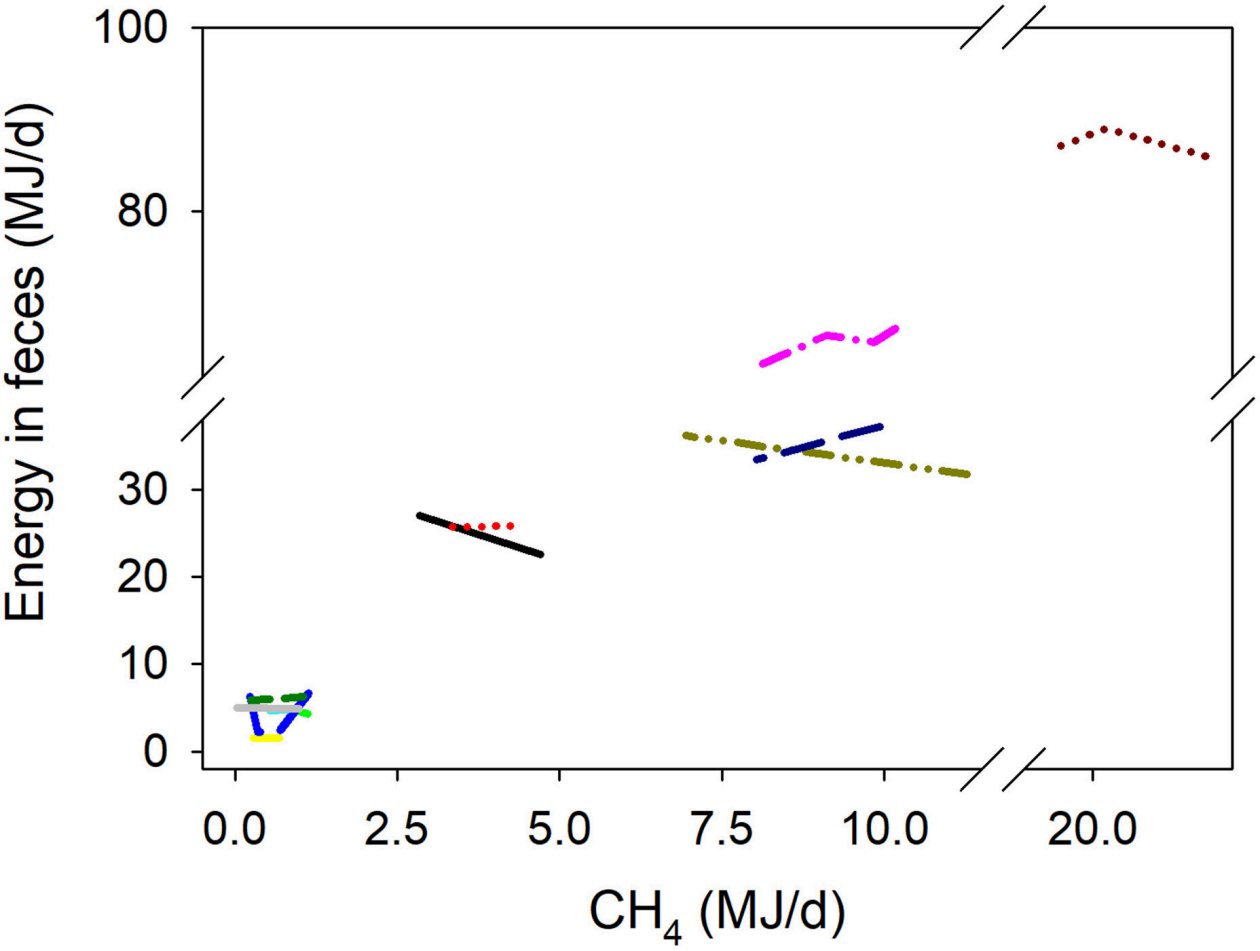
Response of energy losses in feces (EF, MJ/d) adjusted by gross energy intake (GEI, MJ/d) to methanogenesis inhibition, including the random effect of the experiment nested in the cluster, and the random effect of the cluster. Each separate line corresponds to a different experiment: EF = 0.24 (±1.81; *P* = 0.90) + 0.34 (±0.019; *P* < 0.001) GEI – 0.34 (±0.36; *F* = 0.48, *P* = 0.35) CH_4_ – 0.088 (±0.018; *F* = 15.8, *P* < 0.001) (CH_4_ – 5.12)^2^; *N* = 31, 12 experiments.

**Figure 5:**
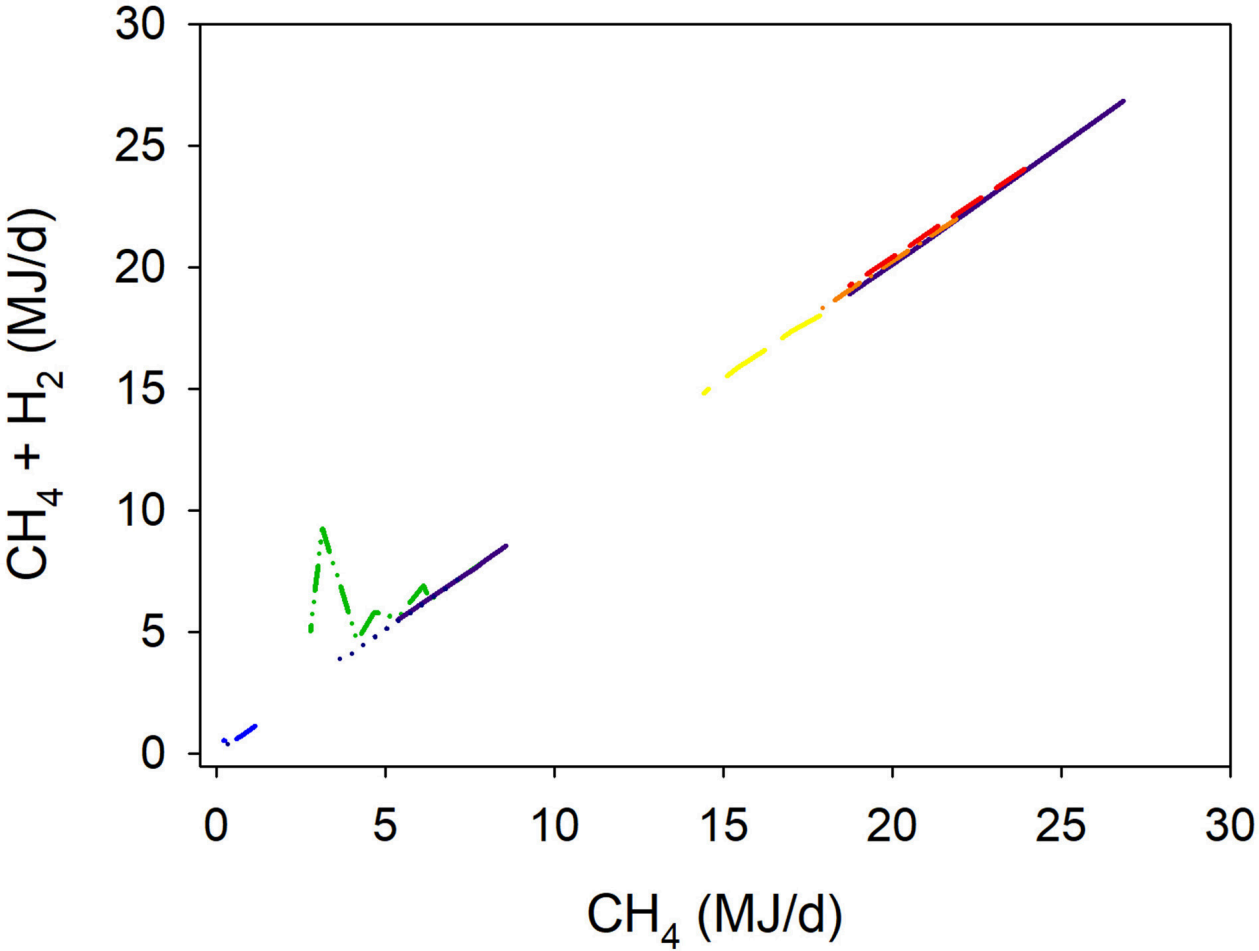
Response of energy losses in gases (CH_4_ + H_2_, EG, MJ/d) adjusted by gross energy intake (GEI, MJ/d) to methanogenesis inhibition, including the random effect of the experiment nested in the cluster, and the random effect of the cluster. Each separate line corresponds to a different experiment: EG = 0.85 (±0.64; *P* = 0.28) + 0.0013 (±0.0030; *P* = 0.67) GEI + 0.92 (±0.064; *F* = 208, *P* < 0.001) CH_4_; *N* = 38, 9 experiments.

**Figure 6:**
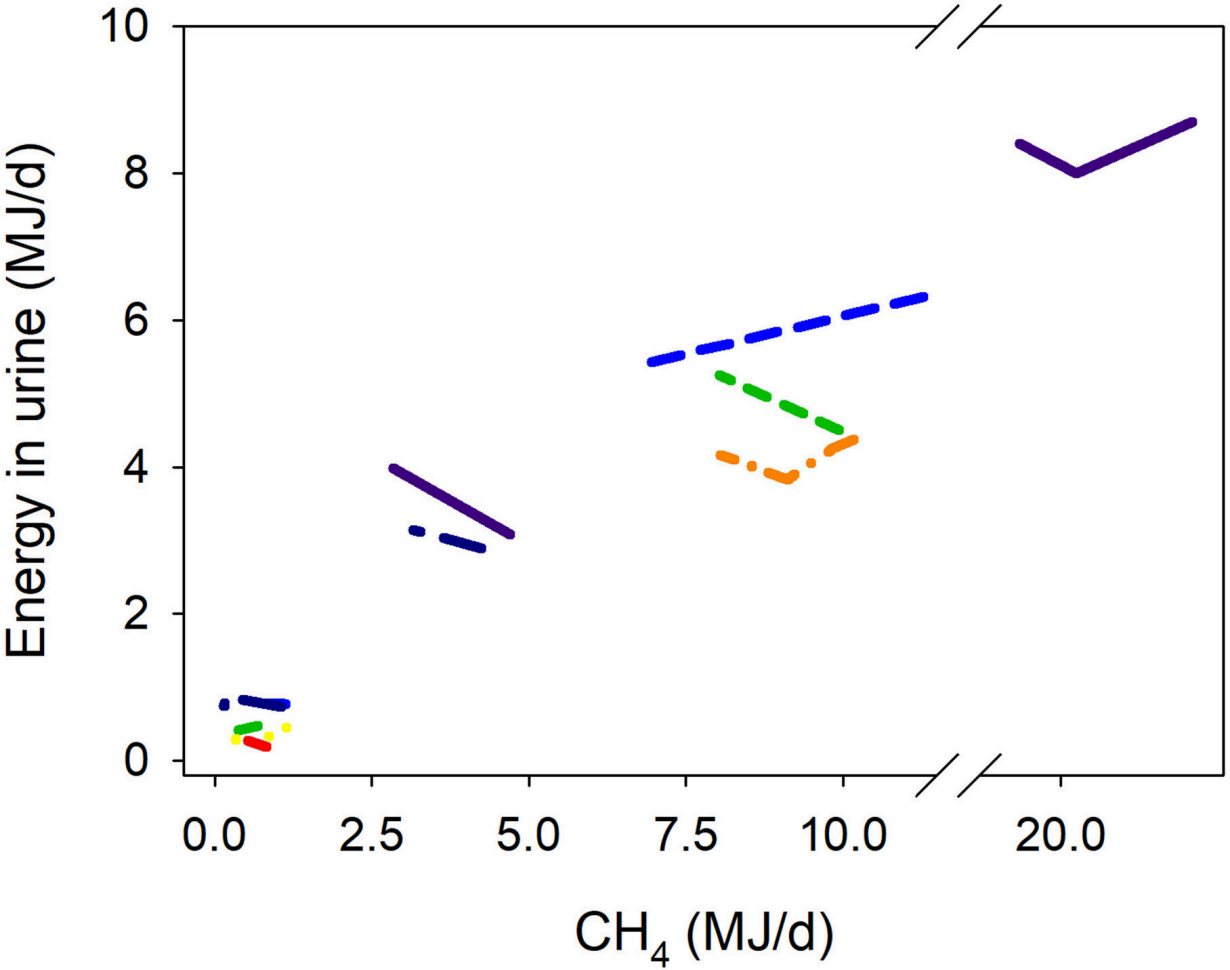
Response of energy losses in urine (UE, MJ/d) adjusted by gross energy intake (GEI, MJ/d) to methanogenesis inhibition, including the random effect of the experiment nested in the cluster, and the random effect of the cluster. Each separate line corresponds to a different experiment: UE = 1.02 (±0.77; *P* = 0.29) + 0.018 (±0.0049; *P* < 0.001) GEI – 0.042 (±0.070; *F* = 0.37, *P* = 0.55) CH_4_; *N* = 29, 11 experiments.

**Figure 7:**
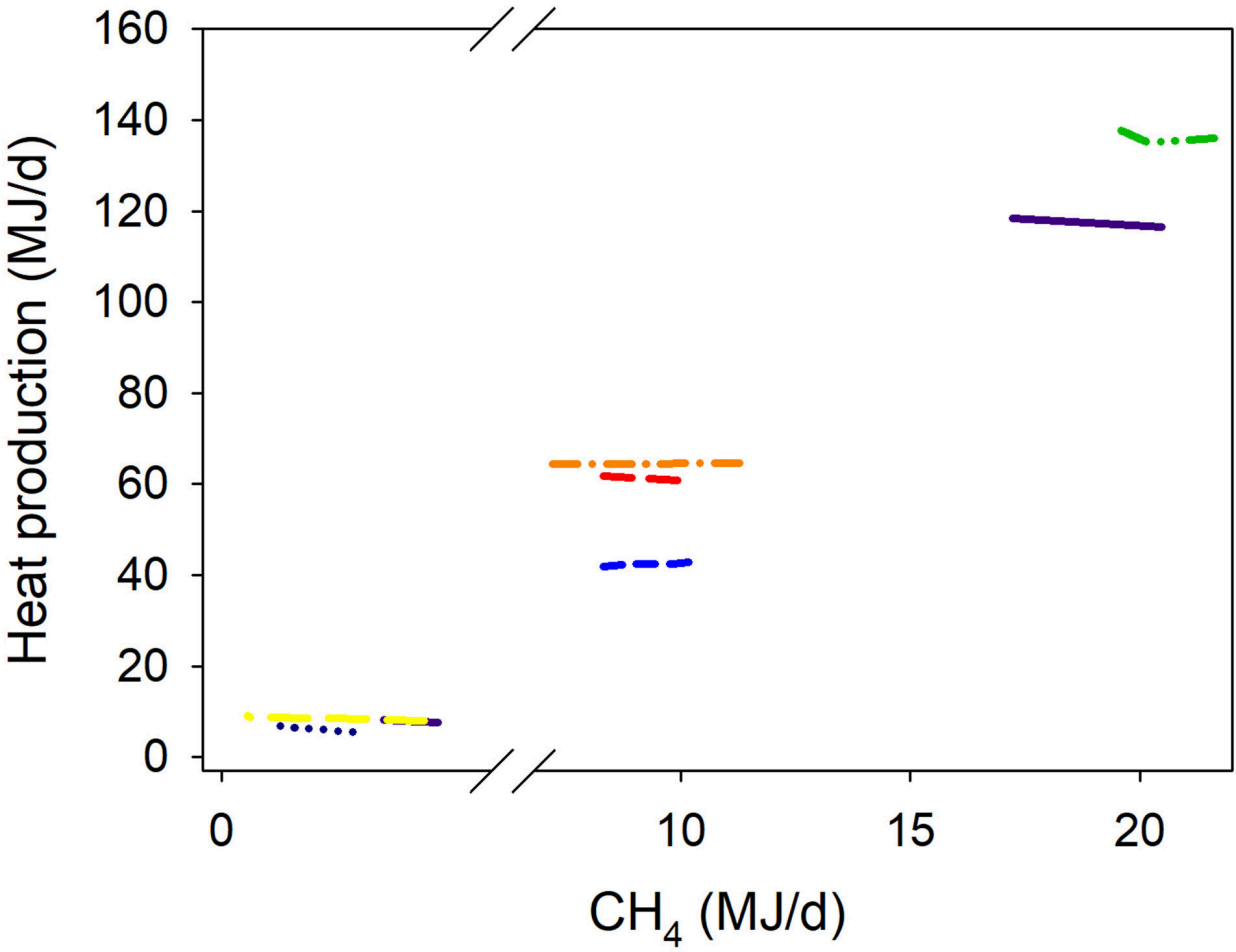
Response of heat production (HP, MJ/d) adjusted by gross energy intake (GEI, MJ/d) to methanogenesis inhibition, including the random effect of the experiment nested in the cluster, and the random effect of the cluster. Each separate line corresponds to a different experiment: HP = 2.27 (±11.6; *P* = 0.86) + 0.36 (±0.042; *P* < 0.001) GEI – 0.011 (±0.011; *F* = 1.04, *P* = 0.33) CH_4_; *N* = 21, 8 experiments.

There was a quadratic (*P* < 0.001) negative association between methanogenesis inhibition and energy output in feces adjusted by GEI (Figure 4). Inhibition of rumen methanogenesis negatively associated with total energy losses in gases (CH_4_ plus H_2_) (*P* < 0.001; Figure 5). For each MJ saved in CH_4_, there was a numerical increase of 0.027 MJ in energy losses as H_2_ (*P* = 0.13; not shown). There were no relationships between inhibition of rumen methanogenesis and energy losses in urine (*P* = 0.55; Figure 6) or heat (*P* = 0.33; Figure 7).

### Rumen variables

Inhibiting rumen methanogenesis was positively associated with rumen pH (*P* = 0.023), and had a negative, quadratic (*P* = 0.056) relationship with total VFA concentration (*P* = 0.002; Table 3). Acetate concentration associated negatively with methanogenesis inhibition (*P* < 0.001; Table 3).

**Table 3:** Effects of inhibiting methanogenesis on rumen variables.

**Equation^a^**	***N***	**Number of experiments**
pH = 6.66 (±0.12; *P* < 0.001) – 0.00074 (±0.00031; *F* = 5.70, *P* = 0.023) CH_4_	50	17
Total VFA^b^ (mM) = 80.1 (±7.06; *P* < 0.001) + 0.059 (±0.019; *F* = 9.64, *P* = 0.023) CH_4_ – 7.84 × 10^−5^ (±4.04 × 10^−5^; F = 3.77, *P* = 0.056) (CH_4_ – 192)^2^	86	30
Acetate (mM) = 48.0 (±4.60; *P* < 0.001) + 0.038 (±0.0099; *F* = 14.7, *P* < 0.001) CH_4_	86	30
Propionate (mM) = 19.5 (±1.86; *P* < 0.001) – 0.0029 (±0.0045; *F* = 0.42, *P* = 0.53) CH_4_	86	30
Butyrate (mM) = 11.9 (±1.31; *P* < 0.001) – 0.0021 (±0.0022; *F* = 0.91, *P* = 0.34) CH_4_	86	30
Isobutyrate (mM) = 1.72 (±0.40; *P* = 0.006) – 0.00040 (±0.00050; *F* = 0.64, *P* = 0.43) CH_4_	54	18
Valerate (mM) = 2.07 (±0.31; *P* < 0.001) – 0.0011 (±0.00053; *F* = 2.08, *P* = 0.048) CH_4_	63	22
Isovalerate (mM) = 2.30 (±0.25; *P* < 0.001) – 0.0021 (±0.00082; *F* = 2.56, *P* = 0.013) CH_4_	59	20
${\text{NH}}_{4}^{+}$ (mM) = 11.4 (±3.87; *P* = 0.026) + 0.0047 (±0.0029; *F* = 2.63, *P* = 0.11) CH_4_	65	22
Total bacteria (log_10_ 16S rRNA gene copies) = 10.6 (±0.77; *P* < 0.001) – 0.00033 (±0.0011; *F* = 0.090, *P* = 0.77) CH_4_	27	10
Total protozoa (log_10_ 18S rRNA gene copies) = 6.93 (±1.53; *P* = 0.17) – 0.00036 (±0.00043; *F* = 0.70, *P* = 0.42) CH_4_	25	9
Total methanogens (log_10_ 16S rRNA gene copies or log_10_ 16S *mcrA* gene copies) = 7.75 (±1.39; *P* < 0.001) + 0.0015 (±0.00051; *F* = 8.65, *P* = 0.009) CH_4_	27	10

a *All regression models include the random effect of the experiment nested in the cluster and the random effect of the cluster*.

b *CH_4_, (L/d); ${\text{NH}}_{4}^{+}$, ammonium; VFA, volatile fatty acids*.

There were no relationships between the inhibition of rumen methanogenesis and the concentration of propionate (*P* = 0.53), butyrate (*P* = 0.34), and isobutyrate (*P* = 0.43; Table 3). There was a positive relationship between the inhibition of rumen methanogenesis and valerate concentration (*P* = 0.048; Table 3). A sensitivity analysis found no relationship between methanogenesis inhibition and valerate concentration if eliminating the experiments by El-Zaiat et al. (47) (*P* = 0.34) or Haisan et al. (48) (*P* = 0.14; not shown). Inhibiting methanogenesis associated with increased concentration of isovalerate (*P* = 0.013; Table 3). There was no relationship between methanogenesis inhibition and rumen ammonium concentration (*P* = 0.11; Table 3).

There was no relationship between inhibition of rumen methanogenesis and total bacterial *16S rRNA* gene copies (*P* = 0.77) or total protozoal *18S rRNA* gene copies (*P* = 0.42; Table 3). There was a negative relationship between methanogenic Archaea *16S rRNA* and *mcrA* gene copies and methanogenesis inhibition (*P* = 0.009; Table 3).

## Discussion

### Animal performance

Inhibiting rumen methanogenesis saves energy otherwise lost as CH_4_ and would theoretically result in greater efficiency of energy utilization. In the present analysis, inhibiting rumen methanogenesis tended to positively associate with improved milk production efficiency when treatment means were weighted by the reciprocal of their SEM. The fact that this tendency found could change toward significance or non-significance by omitting or weighting particular experiments indicates the need for more experiments to obtain more consistent conclusions.

The expected ECM production adjusted by DMI at a theoretical 100% inhibition of methanogenesis would be 3.56 ± 1.97 kg/d greater than the mean of the methanogenesis-uninhibited control treatments, representing 11.2 ± 6.19 MJ. The average CH_4_ production of the control treatments of the ECM analysis was of 532 L/d, equivalent to 19.4 MJ, which would be saved at a theoretical 100% methanogenesis. At the same time, the predicted energy losses as H_2_ at 100% methanogenesis inhibition would have augmented by 1.27 MJ/d, resulting in about 18.1 MJ/d of energy saved in gases. Therefore, the energy saved in gases not produced would be transferred to milk with an efficiency of approximately 62% (calculations not shown). Energy saved in gases becomes metabolizable energy (ME). Conversion of ME to net energy (NE) has been reported to be of 64% (77), which compares well with the 62% figure obtained herein.

Similarly to milk production efficiency, benefits of inhibiting rumen methanogenesis on growth and fattening efficiency also depended on whether treatment means were weighted by the reciprocal of their SEM, with the added complexity that SEM were available for 11 out of the 13 DMI-adjusted BMG experiments. Thus, part of the discrepancy between the analyses with weighted or non-weighted treatment means is due to the omission of the experiments by Davies et al. (29) and Tomkins et al. (36) with weighted treatment means. Again, the influence of the presence and weight of individual experiments on the results also indicates the need for more research on the response of growth and fattening to the inhibition of rumen methanogenesis.

Inhibition of CH_4_ production in the rumen cannot be considered as an isolated intervention and can potentially have consequences both on products of rumen fermentation other than CH_4_ and on post-absorptive metabolism. The discussion that follows examines whether inhibiting methanogenesis could have altered energy losses in the rumen or post-ruminally, and net energy partition.

### Energy losses

The positive association between energy output in feces and the inhibition of rumen methanogenesis does not agree with the lack of relationship observed in the fecal output of DM, OM, N, and NDF adjusted by the corresponding intakes of those fractions. Part of the discrepancy between the relationships in the fecal output of energy on the one hand and DM, OM, N, and NDF on the other hand, with methanogenesis inhibition, is due to differences in the subset of experiments used for each analysis, as different experiments reported digestibility of different fractions. This highlights the importance of conducting more methanogenesis inhibition experiments simultaneously determining the responses in the digestibilities of DM, OM, N, NDF, and energy. In particular, there is much scarcity of determinations of energy digestibility in milk production experiments, as in the only milk production experiment in which energy digestibility was reported methanogenesis inhibition was <10% of the control treatment (52). The lack of relationships between the output of DM and OM in feces and methanogenesis inhibition found herein largely agrees with *in situ* experiments (37, 54), although one cannot rule out that if decreases in rumen digestibility occurred in some experiments they were somewhat compensated by post-ruminal digestion.

In the present analysis, rumen VFA concentration had a negative relationship with methanogenesis inhibition. *In vivo* VFA concentration is not an accurate proxy of fermentative activity, as it does not consider changes in rumen volume (78) and in VFA rates of absorption (79, 80). In *in vitro* batch and continuous cultures, in which actual VFA production can be measured, inhibiting methanogenesis resulted in a decrease in estimated fermented hexoses calculated from the VFA production stoichiometry, and generally a decrease in enthalpy in total VFA (81).

The increase in energy losses as H_2_ in relation to energy saved in CH_4_ not produced varied widely across experiments and was on average much lower than energy saved in CH_4_. Importantly, in some experiments in which the energy gain obtained from inhibiting CH_4_ production surpassed the energy losses as H_2_ there was still no increase in productivity associated (25, 26, 56, 60, 62). Thus, the increase in energy losses as H_2_ does not seem to account for, at least as the only explanation, the lack of consistent improvements in ruminant productivity associated to rumen methanogenesis inhibition. That said, the addition of phloroglucinol to the methanogenesis-inhibited rumens of steers, which partially relieve H_2_ accumulation by approximately 50%, translated into greater bodymass gain of steers (66).

Energy losses in urine and heat were unrelated to rumen methanogenesis inhibition and therefore seem unlikely to explain the lack of consistent positive relationships between ruminant productivity and the decrease in energy losses as CH_4_, although, the same as with other response variables, more experiments are needed to estimate relationships between methanogenesis inhibition and energy losses in urine and heat more accurately.

### Net energy partition

Less DMI with methanogenesis inhibition could imply decreased NE intake and increased proportion of NE utilized for maintenance, leaving less NE available for production purposes, i.e., the opposite of the dilution of maintenance effect (82). It would therefore be important to better understand the relationship between the inhibition of rumen methanogenesis and DMI. Decrease in DMI can take place through retarding rumen particle outflow i.e., physical filling (83). Inhibiting methanogenesis with nitrate resulted in no changes in rumen fluid outflow rate or volume, but DMI was not affected in that experiment (37). The effect of inhibiting rumen methanogenesis on the outflow rate of rumen particles has not been reported to the author's knowledge.

Intake could also be controlled metabolically through the flow of absorbed propionate, which stimulates the oxidation of acetyl-CoA in the liver, and may act as a satiety signal (83). Inhibiting methanogenesis did not relate to propionate concentration in the present analysis. However, if treatments using nitrate, whose reduction is estimated to be thermodynamically very favorable in the rumen (84) and thus competes with propionate formation for reducing equivalents, were not considered, inhibiting methanogenesis associated positively with propionate concentration (*P* = 0.037; not shown). Nevertheless, it is unknown if the actual flows of propionate production and absorption are affected by inhibiting CH_4_ formation in the rumen; the effects of inhibiting rumen methanogenesis on the flows of VFA production and absorption are identified as a gap in knowledge necessary to understand, and perhaps if possible intervene or manage, if inhibiting rumen CH_4_ production is to translate into benefits in animal productivity.

Potentially poor palatability caused by chemical inhibitors of methanogenesis is not thought to have a general effect on intake due to the variation among products in their chemical nature.

Net energy for production can be utilized in various biosynthetic processes. Lactating animals can direct an important proportion of NE for milk production. However, in mid and late lactation, part of NE for production is used to replenish adipose tissue mobilized in early lactation. In the experiments by Haisan et al. (48), Hristov et al. (56), and Haisan et al. (65), although no productivity gain was obtained in terms of energy output in milk, animals in methanogenesis-inhibited treatments either tended to gain, or numerically gained more bodymass than control animals. In fact, if the extra bodymass gained corresponded entirely to adipose tissue, and assuming a heat of combustion of 32.2 MJ per kilogram of adipose tissue (85), the energy gained in bodymass accretion would surpass the energy saved in CH_4_ emissions in the experiment by Haisan et al. (48), and would be roughly comparable to the energy saved in gases emissions in the experiment by Hristov et al. (56). Haisan et al. (65) arrived at similar conclusions estimating energy expenditure in numerical extra bodymass gain with methanogenesis inhibition. Conversely, in the experiment by Abecia et al. (42), in which inhibiting rumen methanogenesis resulted in improved energy output in milk per kilogram DMI, there were no associated differences in bodymass changes.

At present, the number of experiments reporting changes in bodymass and body condition score in lactating animals is insufficient to arrive at solid conclusions about the potential importance of changes in NE partition and replenishment of body reserves that might be induced by rumen methanogenesis inhibition. Furthermore, changes in bodymass and body condition score may not reflect energy balance with accuracy. The replenishment of body reserves of lactating ruminants has important implications to reproductive function efficiency and metabolic diseases such as ketosis (86–88). To the knowledge of this author, the long-term effects of inhibiting rumen methanogenesis on reproductive efficiency and metabolic diseases have not been evaluated.

Likewise, in growing and fattening animals, body mass accretion can occur as different proportions of lean and adipose tissue, which has profound implications to the energy content of gained bodymass. Tomkins et al. (36) found a numerical increase in P8 fat depth in the carcass of steers fed a methanogenesis inhibitor. The same as with milk production, information is very scarce about the possible effects of inhibiting methanogenesis on NE partition, including the partition of NE for gain among accretion of different tissues.

### Other aspects

It is also possible that methanogenesis inhibition simply needs to be more extreme for its energy saving effects to be more consistent. The maximal inhibition of methanogenesis in milk production and in growth and fattening experiments in the present database was on average of 28 and 48%, respectively (not shown). There was only one milk production experiment in which CH_4_ production was inhibited by 60%, although there were no effects on ECM or ECM per kg DMI (48). It is tempting to think that the sheer greater extent of methanogenesis inhibition in some growth and fattening experiments might have resulted in the productivity gains observed (31, 36).

The potential consequences of changes in the acetate to propionate production ratio that might occur on NE allocation toward milk production or body reserves have been discussed (89) but *in vivo* experimental work on the effects of methanogenesis inhibition on acetate and propionate flows is missing. Furthermore, diets used in methanogenesis inhibition experiments are formulated to match nutrient requirements. If inhibiting CH_4_ production augments the flow of absorbed propionate, some decrease in glucogenic precursors supplied by the basal diet may be needed for the extra supply of glucogenic precursors resulting from inhibiting methanogenesis to consistently benefit animal production.

## Final remarks

Gains in productivity appear as important for adoption of CH_4_-mitigation techniques by producers. However, a consistent association between the inhibition of rumen methanogenesis and improvements in ruminant productivity could not be confirmed by the present research. The reader is cautioned about the limitations of the present analysis in terms of number of experiments and uniformity of the response variables reported in them. Also, some of the older experiments used few animals and estimates of variation were not reported.

Capitalizing energy savings in CH_4_ not formed as animal productivity may require refinements of the methanogenesis inhibition intervention, such as modifications of basal diets and/or combinations with other interventions. Development of rational strategies to translate methanogenesis inhibition into gains in productivity will likely require a more complete understanding of some existing gaps in knowledge such as the effects of inhibiting CH_4_ production in the rumen on digestion and on the flows of VFA production and absorption, the mechanisms through which inhibiting methanogenesis affects DMI, and effects on NE partition. At the same time, more performance experiments both on milk production and growth and fattening are needed, including treatments targeting both moderate and severe methanogenesis inhibition.

## Ethics statement

This study is a meta-analysis of published literature. It did not involve any experimental work. It involves summarization of published work and mathematical modeling.

## Author contributions

EU conceived the research, compiled the database, conducted the meta-analysis, interpreted the results and wrote and edited the manuscript.

### Conflict of interest statement

The author declares that the research was conducted in the absence of any commercial or financial relationships that could be construed as a potential conflict of interest.

## Abbreviations

BMG: bodymass gain
CH_4_: methane
CO_2_: carbon dioxide
DE: digestible energy
DM: dry matter
DMD: apparent digestibility of dry matter
DMI: dry matter intake
ECM: energy-corrected milk
GE: gross energy
GEI: gross energy intake
[H]: metabolic hydrogen
H_2_: dihydrogen
HP: heat production
ME: metabolizable energy
ND: apparent digestibility of nitrogen
NDFD: neutral detergent fiber digestibility
NE: net energy
NE_m_: net energy for maintenance
OM: organic matter
OMD: apparent digestibility of organic matter
SEM: standard error of the mean
UE: urine energy
VFA: volatile fatty acids.
