# Supplementary material for: Inhibition of Rumen Methanogenesis and Ruminant Productivity: A Meta-Analysis
Source: Front Vet Sci. 2018 Jun 19;5:113. doi: 10.3389/fvets.2018.00113 (PMC6018482; doi:10.3389/fvets.2018.00113)
Supplement: Supplementary file 1 [file Table_1.docx]

Supplementary Material

**Consequences of the Inhibition of Rumen Methanogenesis on Ruminant Productivity: a Meta-Analysis**

*** Correspondence:** Corresponding Author: emilio.ungerfeld@inia.cl

**Table S1**. Summary of methanogenesis inhibition experiments employing pure chemical additives used in the meta-analysis.

| Reference | Response variables reported^1^ | Animal | Experiment design^2^ | Type of diet^3^ | Methanogenesis inhibitor | Comments |
| --- | --- | --- | --- | --- | --- | --- |
| Czerkawski et al. (1) | ED, UE, HP | Sheep | Treatment switch  (n = 1) | Roughage | Sodium laurate sulfate |  |
| Clapperton and Czerkawski (2), Experiment Series A | DMD, OMD, ND, ED, UE | Sheep | Treatment switch  (n = 2) | Roughage | Tertiary branched-carboxylic acids | SEM not provided |
| Clapperton and Czerkawski (2), Experiment Series B | DMD, OMD, ND, ED, UE |  |  | Mixed |  |  |
| Clapperton and Czerkawski (2), Experiment Series C | OMD, ED, UE |  |  | Mixed |  |  |
| Johnson (3) | H_2_, ED, UE | Sheep | Fixed assignment to treatments (n = 4) | Mixed | Hemiacetal of chloral and starch |  |
| Johnson (4) | H_2_, ND, ED, UE, HP | Sheep | Fixed assignment to treatments (n = 6) | High-concentrate | Hemiacetal of chloral and starch | Only measurement at 3 d post-treatment. Measurement at 30 d post-treatment not considered to avoid dependence |
| Sawyer et al. (5) | DMD, ND, ED, UE, HP, rumen pH and VFA | Sheep | Treatment switch  (n = 4) | Ingredients content not provided | BCM | SEM not provided |
| Cole and McCroskey (6) Experiment 1 | ED, UE | steers | Fixed assignment to treatments (n = 6) | High-concentrate | Hemiacetal of chloral and starch |  |
| Cole and McCroskey (6) Experiment 2 | ED, UE | steers | Fixed assignment to treatments (n = 6) | High-concentrate | Hemiacetal of chloral and starch |  |
| Davies et al. (7) | DMI, BMG, GFE | steers | Fixed assignment to treatments (n = 2) | High-concentrate | Compound ICI 13409 (developed by private company) | SEM not provided |
| Mathers and Miller (8) Experiment 1 | DMD, OMD, ND, ED, rumen pH, VFA, NH_4_^+^ | sheep | Treatment switch  (n = 4) | Mixed | Chloral hydrate |  |
| McCrabb et al. (9) Experiment 1 | DMI, BMG | steers | Fixed assignment to treatments (n = 3) | Roughage | BCM |  |
| Lila et al. (10) | DMI, DMD, ND, NDFD, Rumen pH, VFA, NH_4_^+^ | steers | Treatment switch  (n = 4) | mixed | Diallyl maleate |  |
| Mohammed et al. (11) | DMD, ND, NDFD, rumen pH, VFA, NH_4_^+^ | steers | Treatment switch  (n = 4) | Mixed | Iodopropane |  |
| Tomkins and Hunter (12) | DMI | steers | Fixed assignment to treatments (n = 4) | Not provided | BCM |  |
| Denman et al. (13) | DMI, BMG, VFA | steers | Treatment switch  (n = 6) | Roughage | BCM |  |
| Tomkins et al. (14) Experiment 2 | DMI, BMG | steers | Fixed assignment to treatments (n = 5) | roughage | BCM | Average of 30, 60 and 90 d determinations |
| Nolan et al. (15) | DMD, rumen pH, VFA, NH_4_^+^ | sheep | Fixed assignment to treatments (n = 4) | roughage | nitrate |  |
| van Zijderveld et al. (16) | rumen VFA, NH_4_^+^, microbial groups | Sheep | Fixed assignment to treatments (n = 5) | roughage | Nitrate and sulfate |  |
| Brown et al. (17) | DMI, rumen VFA | steers | Fixed assignment to treatments (n = 6) | roughage | nitroethane |  |
| van Zijderveld et al. (18) | Milk production and composition, NDFD, HP | Dairy cows | Fixed assignment to treatments (n = 10) | mixed | nitrate |  |
| Mitsumori et al. (19) | DMD, OMD, NDFD, H_2_, ED, rumen VFA | goats | Treatment switch  (n = 3) | mixed | BCM | Intake and digestibility provided for control and high inhibition treatments only |
| Abecia et al. (20) | DMI, milk production and composition, rumen VFA, microbial groups | goats | Fixed assignment to treatments (n = 9) | mixed | BCM |  |
| Hulshof et al. (21) | DMI, rumen VFA, NH_4_^+^ | steers | Treatment switch  (n = 8) | mixed | nitrate |  |
| Li et al. (22) | DMI, BMG | lambs | Fixed assignment to treatments (n = 5) | Not provided | nitrate |  |
| Shinkai et al. (23) Experiment 1 | DMD, OMD, ND, NDFD, ED, UE, HP, rumen pH, VFA, NH_4_^+^ | cows | Treatment switch  (n = 3) | mixed | cashew nut shell |  |
| Shinkai (23) Experiment 2 |  |  |  |  |  |  |
| Martinez-Fernandez et al. (24) Experiment 3, propyl propane treatments (Experiments 1 and 2 were *in vitro*) | DMI | goats | Fixed assignment to treatments (n = 4) | mixed | propyl propane thiosulfinate or BCM | Treatments with propyl propane thiosulfinate and BCM had each their own controls and were thus treated as different experiments |
| Martinez-Fernandez (24) Experiment 3, BCM treatments (Experiments 1 and 2 were *in vitro*) |  |  |  |  |  |  |
| El-Zaiat et al. (25) | DMI, BMG, rumen VFA, NH_4_^+^ | sheep | Fixed assignment to treatments (n = 6) | mixed | Nitrate and encapsulated nut shell |  |
| Haisan et al. (26) | DMI, milk production and composition, rumen VFA, microbial groups | Dairy cows | Treatment switch  (n = 12) | mixed | NOP |  |
| Lund et al. (27) | DMI, H_2_ | Dairy cows | Treatment switch  (n = 4) | mixed | nitrate |  |
| Newbold et al. (28) Experiment 1 | DMI | steers | Fixed assignment to treatments (n = 6) | High concentrate | nitrate |  |
| de Raphélis-Soissan et al. (29) | BMG, rumen pH, VFA, NH_4_^+^ | sheep | Fixed assignment to treatments (n = 7) | roughage | nitrate |  |
| Reynolds et al. (30) | DMI, milk production and composition, DMD, OMD, NDFD, ED, UE, HP, rumen pH, VFA, NH_4_^+^ | Dairy cows | Treatment switch  (n = 6) | mixed | NOP | Methanogenesis inhibition was weak |
| Romero-Perez et al. (31) | DMI, BMG, DMD, OMD, ND, NDFD, ED, rumen pH, VFA, NH_4_^+^, microbial groups | Beef heifers | Treatment switch  (n = 8) | mixed | NOP |  |
| Martínez-Fernández et al. (32) Experiment 2 | rumen VFA, NH_4_^+^, microbial groups | Sheep | Treatment switch  (n = 6) | Mixed | ethyl-3-nitrooxy propionate | BMG and GFE results excluded because animals were fed at maintenance |
| Martínez-Fernández (32) Experiment 3 |  |  | Treatment switch  (n = 9) |  | NOP and ethyl-3-nitrooxy propionate |  |
| Mitsumori et al. (33) Experiment 1 | Rumen VFA | cows | Treatment switch  (n = 3) | mixed | Cashew nut shell |  |
| Mitsumori et al. (34) Experiment 2 |  |  |  |  |  |  |
| Hristov et al. (35) | DMI, H_2_, milk production and composition, DMD, OMD, ND, NDFD | Dairy cows | Fixed assignment to treatments (n = 12) | mixed | NOP |  |
| Lee et al. (36) | DMI, BMG, DMD, OMD, NDFD, ED, UE, HP, N retention, rumen NH_4_^+^ | Beef heifers | Fixed assignment to treatments (n = 8) | mixed | NOP |  |
| Romero-Perez et al. (37) | Rumen pH, VFA, NH_4_^+^, microbial groups | Beef heifers | Fixed assignment to treatments (n = 8) | mixed | NOP | Animals at maintenance |
| Veneman et al. (38) Experiment 1 | DMI, H_2_, milk production and composition, rumen VFA, NH_4_^+^, microbial groups | Dairy cows | Fixed assignment to treatments (n = 6) | mixed | nitrate | Linseed treatment excluded because of greater energy content |
| Veneman (38) Experiment 2 | DMI, H_2_, milk production and composition, rumen VFA, NH_4_^+^, microbial groups | Dairy cows | Fixed assignment to treatments (n = 6) | mixed | nitrate | Linseed treatment excluded because of greater energy content |
| Lopes et al. (39) | DMI, H_2_, milk production and composition, MPE, rumen pH, VFA, NH_4_^+^, microbial groups | Dairy cows | Treatment switch  (n = 6) | mixed | NOP |  |
| Martinez-Fernandez et al. (40) | DMI, H_2_, rumen pH, VFA, NH_4_^+^ | Steers | Treatment switch  (n =4) | roughage | chloroform | 1. Performance data for mixed diet low, mid and high chloroform supplementation provided courtesy of G. Martínez-Fernández |
|  |  |  |  | mixed |  |  |
| Olijhoek et al. (41) | DMI, H_2_, milk production and composition, DMD, OMD, ND, NDFD, rumen pH, VFA, NH_4_^+^ | Dairy cows | Treatment switch  (n = 4) | mixed | nitrate |  |
| Vyas et al. (42) Experiment 1 | DMI, H_2_, BMG | steers | Fixed assignment to treatments (n = 28) | mixed | NOP |  |
| Vyas (42) Experiment 2 |  |  |  |  |  |  |
| Vyas et al. (43) Experiment 1 | DMI, H_2_ | steers | Treatment switch  (n = 5) | mixed | NOP |  |
| Vyas (43) Experiment 2 |  |  |  |  |  |  |
| Haisan et al. (44) | DMI, milk production and composition, DMD, OMD, ND, NDFD, rumen pH, VFA, NH_4_^+^ | Dairy cows | Treatment switch  (n = 15) | mixed | NOP |  |
| Martinez-Fernandez et al. (45) | DMI, H_2_, rumen pH, VFA, NH_4_^+^ | steers | Treatment switch  (n = 8) | Mixed | chloroform | control period *vs.* chloroform period |

^1^Abbreviations: BCM = bromochloromethane; DMD = apparent dry matter digestibility; DMI = dry matter intake; ED = apparent energy digestibility; H_2_ = dihydrogen production (L/d); HP = heat production (%gross energy intake); ND = apparent nitrogen digestibility; NDFD = neutral detergent fiber digestibility; NH_4_^+^ = ammonium concentration; NOP = 3-nitrooxypropanol; OMD = apparent organic matter digestibility; UE = urine energy (%gross energy intake); VFA = volatile fatty acids concentration and individual molar percentage.

^2^Fixed assignment to treatments include random plots and random block experiments. Treatment switch include cross over and Latin Square designs and designs with an initial control period followed by one or more treatment periods.

^3^Roughage: <⅓ dry matter (DM) as concentrate; mixed: between ⅓ and ⅔ DM as concentrate; high-concentrate: >⅔ DM as concentrate.

**Table S2**. Summary of methanogenesis inhibition experiments employing pure chemical additives not used in the meta-analysis.

| Reference | Response variables reported or calculated^1^ | Animal | Experiment design^2^ | Type of diet^3^ | Methanogenesis inhibitor | Comments |
| --- | --- | --- | --- | --- | --- | --- |
| van Nevel et al. (46) | Rumen pH, VFA | Sheep | Treatment switch | mixed | Chloral hydrate | Rumen gas collected only through cannula during 8 h/d |
| Farra and Satter (47) (three experiments) | DMI, bodymass, milk production and composition, rumen VFA, NH_4_^+^ | Dairy cows | Fixed assignment to treatments and treatment switch | High concentrate | nitrate | CH_4_ production not provided |
| Trei et al. (48) | DMI, BMG, GFE, rumen VFA, NH_4_^+^ | Sheep | Fixed assignment to treatments | Mixed | Trichloroacetamide | 1. Gas composition of rumen headspace, but not gas production, was provided; 2. SEM not provided |
| Trei et al. (49) | DMI, BMG, GFE | Sheep | Fixed assignment to treatments | Mixed | Hemiacetal of chloral and starch | 1. Gas composition of rumen headspace, but not gas production, was provided;   2) SEM not provided |
| Johnson et al. (50) | DMI, BMG, DMD, ED, rumen VFA | steers | Fixed assignment to treatments | high concentrate | BCM, corn oil, tallow | CH_4_ production provided in graphical form only |
| Lanigan (51) | Rumen gas composition | sheep | Treatment switch | roughage | Chloral hydrate, chloroform, bromoform, iodoform, carbon tertrachloride, carbontetrabromide | Response variables analyzed in the present study not provided |
| Clapperton (52) | DMD, OMD,ND, ED, rumen VFA | sheep | Fixed assignment to treatments | mixed | Trichloroacetamide, chloroform, linseed oil | CH_4_ production provided in graphical form only |
| Czerkawski and Breckenridge (53) (four experiments) | Gas production *in vitro* | sheep | Fixed assignment to treatments | roughage and mixed | Trichloroethyl pivalate and trichloroethyl adipate | Inhibitors were given to sheep, but CH_4_ production was measured *in vitro* with rumen inoculum sampled from the experimental sheep |
| Horton (54) (two experiments) | DMI, BMG, GFE, OMD, ND, rumen VFA, NH_4_^+^ | steers | Fixed assignment to treatments | mixed | Monensin, amicloral | CH_4_ production not reported |
| Horton (55) (two experiments) | DMI, BMG, GFE, OMD, ND, rumen VFA, NH_4_^+^, carcass variables | steers | Fixed assignment to treatments | mixed | Amicloral, 4, 4´-dimethyldiphenyliodonium | CH_4_ production not reported |
| Mathers and Miller (8) Experiment 2 | Rumen pH, VFA, NH_4_^+^ | sheep | Treatment switch | roughage | Chloral hydrate | CH_4_ production not reported |
| McCrabb (9) Experiment 2 | DMI, BMG, BMG/DMI, rumen VFA, NH_4_^+^ | Steers | Fixed assignment to treatments | roughage | BCM | CH_4_ production not reported |
| McCrabb (9) Experiment 3 | DMI, BMG, BMG/DMI, rumen VFA, NH_4_^+^ | Steers | Fixed assignment to treatments | roughage | BCM | CH_4_ production not reported |
| Kung et al. (56) (three experiments) | DMI, BMG, OMD, ND, NDFD, rumen VFA | sheep | Fixed assignment to treatments | Diet ingredients not provided | 9, 10-anthraquinone | CH_4_ production provided in graphical form only and in a different experiment than the rest of the variables |
| Anderson et al. (57) | In vitro variables obtained with rumen inoculum of animals given nitrocompounds | sheep | Fixed assignment to treatments | roughage | nitrocompounds | All variables were determined in *in vitro* cultures with rumen inoculum from treatment animals |
| Gutierrez-Banuelos et al. (58) | Rumen VFA | steers | Fixed assignment to treatments | mixed | Nitrocompounds | CH_4_ production provided in graphical form only |
| Tomkins (14) Experiment 1 | DMI | Steers | Fixed assignment to treatments | High concentrate | BCM | It is not indicated if feeding was *ad libitum* |
| Tomkins (14) Experiment 3 | DMI, BMG, BMG/DMI, carcass variables | steers | Fixed assignment to treatments | High concentrate | BCM | CH_4_ production not reported |
| Knight et al. (59) | DMD, rumen pH, VFA, NH_4_ | cows | Fixed assignment to treatments | roughage | chloroform | CH_4_ production and bodymass provided in graphical form only |
| Klevenhusen et al. (60) | DMI, OMD, NDFD, rumen pH, VFA, NH_4_, microbial groups | Sheep | Treatment switch | mixed | Diallyl disulfide, lovastatin | CH_4_ production not significantly or numerically decreased |
| Lin et al. (61) | Bacterial diversity | steers | Treatment switch | mixed | nitrate | 1. Response variables studied in the present meta-analysis were not provided in this paper; ii) CH_4_ production not reported |
| Abecia et al. (62) | DMI, BMG | goats (kids) | Fixed assignment to treatments | mixed | BCM | 1. DMI provided in a BM^0.75^ basis, but BM at each DMI time point not provided; ii) BMG provided for whole experimental period including pre-weaning |
| Abecia et al. (63) | archaeal diversity | goats (kids) | Fixed assignment to treatments | mixed | BCM | Response variables studied in the present meta-analysis were not provided in this paper |
| Mitsumori (34) | Reductive acetogens diversity | steers | Fixed assignment to treatments | mixed | BCM and fumarate | Response variables studied in the present meta-analysis were not provided in this paper |
| Newbold (28) Experiment 2 | DMI | steers | Fixed assignment to treatments | High concentrate | nitrate | CH_4_ production not reported |
| Asanuma et al. (64) | Rumen pH, VFA, NH_4_^+^ | goats | Treatment switch | mixed | nitrate | CH_4_ production not reported |
| Guyader et al. (65) | DMI, DMD, OMD, NDFD, rumen pH, VFA, NH4+ | Dairy cows | Treatment switch | mixed | nitrate | Nitrate diets contained 3.3 – 4.5% less gross energy |
| Lee et al. (66) | DMI, rumen pH, VFA, NH_4_^+^ | Beef heifers | Treatment switch | mixed | nitrate | CH_4_ production not reported |
| Troy et al. (67) | DMI, H_2_, rumen VFA | steers | Fixed assignment to treatments | Mixed or high concentrate | nitrate | Nitrate replaced a true protein supplement |
| Zhao et al. (68) | Rumen pH, VFA, NH_4_^+^, bacterial diversity | steers | Treatment switch | mixed | nitrate | CH_4_ production not reported |
| Nguyen et al. (69) | DMI, BMG, DMD, rumen pH, VFA, NH_4_^+^ | sheep | Fixed assignment to treatments | roughage | nitrate | Nitrate supplementation increased DMI and BMG (basal diet was N deficient) |
| Guyader et al. (70) | DMI, milk production and composition, DMD, OMD, ND, NDFD, rumen VFA, NH_4_^+^ | Dairy cows | Fixed assignment to treatments | mixed | nitrate | Diet with nitrate also included linseed and was energy-denser |
| Lee et al. (71) | *In situ* N disappearance | Beef heifers | Fixed assignment to treatments | mixed | nitrate | 1. Response variables studied in the present meta-analysis were not provided in this paper; ii) CH_4_ production not reported |

^1^Abbreviations: BCM = bromochloromethane; DMD = apparent dry matter digestibility; DMI = dry matter intake; ED = apparent energy digestibility; H_2_ = dihydrogen production (L/d); HP = heat production (%gross energy intake); ND = apparent nitrogen digestibility; NDFD = neutral detergent fiber digestibility; NH_4_^+^ = ammonium concentration; NOP = 3-nitrooxypropanol; OMD = apparent organic matter digestibility; UE = urine energy (%gross energy intake); VFA = volatile fatty acids concentration and individual molar percentage.

^2^Fixed assignment to treatments include random plots and random block experiments. Treatment switch include cross over and Latin Square designs and designs with an initial control period followed by one or more treatment periods.

^3^Roughage: <⅓ dry matter (DM) as concentrate; mixed: between ⅓ and ⅔ DM as concentrate; high-concentrate: >⅔ DM as concentrate.

**Table S3.** Experimental variables used for clustering experiments in meta-regressions.

| Experimental variable | Levels |
| --- | --- |
| Research center^1^ | Aarhus University, Aberystwyth University, University of Reading, University of New England, University of Maine, University of Illinois, University of Cambridge, University of Alberta, Tokyo University of Agriculture and Technology, Smith Kline & French, Pennsylvania State University, Oklahoma State University, National Institute of Livestock and Grassland Science, Michigan State University, Lethbridge Research Centre (Agriculture and Agri-Feed Canada), Instituto de Zootecnia, Imperial Chemical Industries, Hannah Research Institute, CSIRO Tropical Agriculture, CSIRO Queensland Bioscience Precint, CSIRO JM Rendel Laboratory, AgResearch NZ, Estación Experimental del Zaidín (Consejo Superior de Investigaciones Científicas) |
| Year of publication | 1966 to 2017 |
| Type of experiment design | Fixed assignment of animals to treatments (randomized blocks and plots) or treatment switch (Latin squares and cross overs) |
| Feeding regime | *Ad libitum* or restricted |
| Animal | dairy cows or goats (milk production), steers, heifers or sheep (growth and fattening), steers or sheep (animal physiology experiments with animals at maintenance) |
| Stage of lactation^2^ | Dairy cows: early (0 to 90 d post-parturition), mid (91 to 180 d post-parturition), late (181 to 270 d post-parturition)  Dairy goats: early (0 to 80 d post-parturition), mid (81 to 140 d post-parturition), late (more than 140 d post-parturition) |
| Type of inhibitor | CH_4_ halogenated analogues, chloral hydrate, hemiacetal of chloral and starch, diallyl maleate, versatic acids, trichloroacetamide, nitrate, nitrocompounds, nitrooxycompounds, cashew nut shell, fatty acids sulfate, 2,4-bis(trichloromethyl)-benzo[l,3]dioxin-4-carboxamide |
| Method of CH_4_ production measurement | Chamber, GreenFeed, head hood chamber, SF_6_ gas marker |

^1^Where the experiment was conducted.

^2^Only milk production experiments.

**Table S4.** Clustering of response variables.

| Response | Clusters | Experiments per cluster |
| --- | --- | --- |
| ECM^1^ (kg/d) | 1 | van Zijderveld (18), Abecia (20) |
|  | 2 | Haisan (26), Reynolds (30), Hristov (35), Veneman (38), Lopes (39), Olijhoek (41) |
|  | 3 | Haisan (44) |
| BMG (kg/d) | 1 | Davies (7) |
|  | 2 | McCrabb (9), Denman (13), Tomkins (14), Martinez-Fernandez (40), Martinez-Fernandez (45) |
|  | 3 | Li (22), El-Zaiat (25), de Raphélis-Soissan (29) |
|  | 4 | Romero-Perez (31), Lee (36), Vyas (42) |
| DMI (kg/d) | 1 | Davies (7) |
|  | 2 | Tomkins and Hunter (12), Tomkins (14) |
|  | 3 | Brown (17), Hulshof (21) |
|  | 4 | Li (22), El-Zaiat (25), Lee (36) |
|  | 5 | Romero-Perez (31), Vyas (42), Vyas (43) |
|  | 6 | Martinez-Fernandez (40), Martinez-Fernandez (45) |
|  | 7 | Abecia (20), Reynolds (30), Hristov (35), Lopes (39) |
|  | 8 | Haisan (26), Haisan (44) |
|  | 9 | Lund (27), Veneman (38), Olijhoek (41) |
| DMf (kg/d) | 1 | Clapperton and Czerkawski (2), Sawyer (5), Mathers and Miller (8) |
|  | 2 | Lila (10), Mohammed (11) |
|  | 3 | Mitsumori (19), Shinkai (23), Olijhoek (41) |
|  | 4 | Reynolds (30), Hristov (35), Haisan (44) |
|  | 5 | Nolan (15), Li (22), Romero-Perez (31), Lee (36) |
| OMf (kg/d) | 1 | Mathers and Miller (8) |
|  | 2 | Mitsumori (19), Shinkai (23), Olijhoek (41) |
|  | 3 | Reynolds (30), Hristov (35), Haisan (44) |
|  | 4 | Romero-Perez (31), Lee (36) |
| Nf (kg/d) | 1 | Sawyer (5), Mathers and Miller (8) |
|  | 2 | Lila (10), Mohammed (11) |
|  | 3 | Shinkai (23), Reynolds (30), Romero-Perez (31), Olijhoek (41), Haisan (44) |
|  | 4 | Li (22), Hristov (35) |
| NDFf (kg/d) | 1 | Lila (10), Mohammed (11) |
|  | 2 | van Zijderveld (18), Mitsumori (19), Shinkai (23) |
|  | 3 | Reynolds (30), Romero-Perez (31), Hristov (35), Olijhoek (41), Haisan (44) |
|  | 4 | Lee (36) |
| Energy in feces (MJ/d) | 1 | Czerkawski (1), Johnson (3), Johnson (4), Cole and McCroskey (6) |
|  | 2 | Sawyer (5), Mathers and Miller (8) |
|  | 3 | Shinkai (23), Reynolds (30) |
|  | 4 | Li (22), Lee (36) |
| EG/energy in H_2_ (MJ/d) | 1 | Johnson (3), Johnson (4) |
|  | 2 | Mitsumori (19) |
|  | 3 | Martinez-Fernandez (40), Martinez-Fernandez (45) |
|  | 4 | Hristov (35), Lopes (39) |
|  | 5 | Lund (27), Veneman (38), Olijhoek (41) |
|  | 6 | Vyas (42), Vyas (43) |
| UE (MJ/d) | 1 | Czerkawski (1), Johnson (3), Johnson (4), Sawyer (5), Cole and McCroskey (6) |
|  | 2 | Shinkai (23), Reynolds (30) |
|  | 3 | Li (22), Lee (36) |
| HP (MJ/d) | 1 | Czerkawski (1), Clapperton and Czerkawski (2), Johnson (4), Sawyer (5) |
|  | 2 | van Zijderveld (18), Lee (36) |
|  | 3 | Shinkai (23), Reynolds (30) |
| Rumen pH | 1 | Sawyer (5), Mathers and Miller (8) |
|  | 2 | Lila (10), Mohammed (11) |
|  | 3 | Martinez-Fernandez (40), Martinez-Fernandez (45) |
|  | 4 | Nolan (15), de Raphélis-Soissan (29) |
|  | 5 | Shinkai (23), Lopes (39), Haisan (44) |
|  | 6 | Reynolds (30), Romero-Perez (31), Romero-Perez (37), Veneman (38), Olijhoek (41) |
| Total VFA (mM) | 1 | Sawyer (5) |
|  | 2 | Lila (10), Mohammed (11) |
|  | 3 | Brown (17), Hulshof (21) |
|  | 4 | Denman (13), Mitsumori (19), Abecia (20) |
|  | 5 | Martinez-Fernandez (40), Martinez-Fernandez (45) |
|  | 6 | Shinkai (23), Mitsumori (34) |
|  | 7 | Haisan (26), Reynolds (30), Veneman (38), Lopes (39), Olijhoek (41), Haisan (44) |
|  | 8 | Romero-Perez (31), Martínez-Fernández (32), Romero-Perez (37) |
|  | 9 | Nolan (15), van Zijderveld (16), Li (22), El-Zaiat (25), de Raphélis-Soissan (29) |
| Acetate, propionate, butyrate (mM) | 1 | Sawyer (5), Mathers and Miller (8) |
|  | 2 | Lila (10), Mohammed (11) |
|  | 3 | Brown (17), Hulshof (21) |
|  | 4 | Denman (13), Abecia (20), Martinez-Fernandez (40), Martinez-Fernandez (45) |
|  | 5 | Mitsumori (19), Shinkai (23), Mitsumori (34) |
|  | 6 | Nolan (15), van Zijderveld (16), Li (22), El-Zaiat (25), de Raphélis-Soissan (29), Veneman (38) |
|  | 7 | Haisan (26), Haisan (44) |
|  | 8 | Reynolds (30), Veneman (38), Lopes (39), Olijhoek (41) |
|  | 9 | Romero-Perez (31), Martínez-Fernández (32), Romero-Perez (37) |
| Isobutyrate (mM) | 1 | Sawyer (5) |
|  | 2 | Mohammed (11), Mitsumori (19), Hulshof (21) |
|  | 3 | Martinez-Fernandez (40), Martinez-Fernandez (45) |
|  | 4 | Denman (13), Abecia (20), El-Zaiat (25), Olijhoek (41) |
|  | 5 | Haisan (26), Mitsumori (34), Lopes (39), Haisan (44) |
|  | 6 | Romero-Perez (31), Martínez-Fernández (32), Romero-Perez (37) |
| Valerate (mM) | 1 | Sawyer (5), Mathers and Miller (8) |
|  | 2 | Lila (10), Mohammed (11) |
|  | 3 | Denman (13), Abecia (20), Martinez-Fernandez (40), Martinez-Fernandez (45) |
|  | 4 | Hulshof (21), Haisan (26), Lopes (39), Haisan (44) |
|  | 5 | Mitsumori (34) |
|  | 6 | van Zijderveld (16), El-Zaiat (25), Veneman (38), Olijhoek (41) |
|  | 7 | Romero-Perez (31), Martínez-Fernández (32), Romero-Perez (37) |
| Isovalerate (mM) | 1 | Sawyer (5), Mathers and Miller (8) |
|  | 2 | Lila (10), Mohammed (11) |
|  | 3 | Mitsumori (19), Mitsumori (34) |
|  | 4 | Denman (13), Abecia (20), El-Zaiat (25), Olijhoek (41) |
|  | 5 | Hulshof (21), Haisan (26), Lopes (39), Haisan (44) |
|  | 6 | Martinez-Fernandez (40), Martinez-Fernandez (45) |
|  | 7 | Romero-Perez (31), Martínez-Fernández (32), Romero-Perez (37) |
| NH_4_^+^ (mM) | 1 | Mathers and Miller (8) |
|  | 2 | Mohammed (11), Shinkai (23) |
|  | 3 | Martínez-Fernández (32) |
|  | 4 | Nolan (15), van Zijderveld (16), El-Zaiat (25), de Raphélis-Soissan (29), Veneman (38) |
|  | 5 | Hulshof (21), Lopes (39), Haisan (44) |
|  | 6 | Reynolds (30), Romero-Perez (31), Lee (36), Romero-Perez (37), Veneman (38), Olijhoek (41) |
|  | 7 | Martinez-Fernandez (40), Martinez-Fernandez (45) |
| Bacteria/  methanogens (log_10_ *16S rRNA* or *mcrA* gene copies/g rumen contents) | 1 | van Zijderveld (16), Abecia (20) |
|  | 2 | Haisan (26), Lopes (39), Haisan (44) |
|  | 3 | Romero-Perez (31), Martínez-Fernández (32), Romero-Perez (37), Veneman (38) |
| Protozoa (log_10_ *18S rRNA* gene copies/g rumen contents) | 1 | van Zijderveld (16), Abecia (20) |
|  | 2 | Haisan (26), Haisan (44) |
|  | 3 | Romero-Perez (31), Martínez-Fernández (32), Romero-Perez (37), Veneman (38) |

^1^Abbreviations: BMG = bodymass gain; DMf = dry matter output in feces; DMI = dry matter intake; ECM = energy-corrected milk; EG = energy in gases; HP = heat production; NDFf = NDF output in feces; Nf = nitrogen output in feces; OMf = organic matter output in feces; UE = urine energy; VFA = volatile fatty acids.

**References**

1. Czerkawski JW, Blaxter KL, Wainman FW. The effect of functional groups other than carboxyl on the metabolism of C18 and C12 alkyl compounds by sheep. *Br J Nutr* (1966) **20**:495-508.

2. Clapperton JL, Czerkawski JW. The effect of tertiary branched-chain carboxylic acids on the energy metabolism of sheep. *Brit J Nutr* (1971) **26**:459-68.

3. Johnson DE. Effects of a hemiacetal of chloral and starch on methane production and energy balance of sheep fed a pelleted diet. *J Anim Sci* (1972) **35**:1064-8.

4. Johnson DE. Adaptational responses in nitrogen and energy balance of lambs fed a methane inhibitor. *J Anim Sci* (1974) **38**:154-7.

5. Sawyer MS, Hoover WH, Sniffen CJ. Effect of a ruminal methane inhibitor on growth and energy metabolism in the ovine. *J Anim Sci* (1974) **38**:908-14.

6. Cole NA, McCroskey JE. Effects of hemiacetal of chloral and starch on the performance of beef steers. *J Anim Sci* (1975) **41**:1735-41.

7. Davies A, Nwaonu HN, Stanier G, Boyle FT. Properties of a novel series of inhibitors of rumen methanogenesis; in vitro and in vivo experiments including growth trials on 2,4-bis (trichloromethyl)-benzo [1, 3]dioxin-6-carboxylic acid. *Brit J Nutr* (1982) **47**:565-76.

8. Mathers JC, Miller EL. Some effects of chloral hydrate on rumen fermentation and digestion in sheep. *J Agric Sci (Camb)* (1982) **99**:215-24.

9. McCrabb GJ, Berger KT, T. M, May C, Hunter RA. Inhibiting methane production in Brahman cattle by dietary supplementation with a novel compound and the effects on growth. *Aust J Agr Res* (1997) **48**:323-9. doi: 10.1071/AR96119.

10. Lila ZA, Mohammed N, Tatsuoka N, Kanda S, Kurokawa Y, Itabashi H. Effect of cyclodextrin diallyl maleate on methane production, ruminal fermentation and microbes *in vitro* and *in vivo*. *Anim Sci J* (2004) **75**:15-22.

11. Mohammed N, Lila ZA, Tatsuoka N, Hara K, Mikuni K, Hara K, et al. Effects of cyclodextrin-iodopropane complex on methane production, ruminal fermentation and microbes, digestibility and blood metabolites in steers. (2004) **75**:131-7.

12. Tomkins NW, Hunter RA. Methane reduction in beef cattle using a novel antimethanogen. *Proceedings of the Australian Society of Animal Production 25th Biennial Conference : the new realities*; 2004 4-8th July, 2004 University of Melbourne, Parkville, Vic. Collingwood, Vic.: CSIRO Publishing (2004).

13. Denman SE, Tomkins NW, McSweeney CS. Quantitation and diversity analysis of ruminal methanogenic populations in response to the antimethanogenic compound bromochloromethane. *FEMS Microbiol Ecol* (2007) **62**:313-22. doi: 10.1111/j.1574-6941.2007.00394.x.

14. Tomkins NW, Colegate SM, Hunter RA. A bromochloromethane formulation reduces enteric methanogenesis in cattle fed grain-based diets. *Anim Prod Sci* (2009) **49**:1053-8.

15. Nolan JV, Hegarty RS, Hegarty J, Godwin IR, Woodgate R. Effects of dietary nitrate on fermentation, methane production and digesta kinetics in sheep. *Anim Prod Sci* (2010) **50**:801-6. doi: 1836-0939/10/080801.

16. van Zijderveld SM, Gerrits WJJ, Apajalahti JA, Newbold JR, Dijkstra J, Leng RA, et al. Nitrate and sulfate: effective alternative hydrogen sinks for mitigation of ruminal methane production in sheep. *J Dairy Sci* (2010) **93**:5856-66. doi: 10.3168/jds.2010-3281.

17. Brown EG, Anderson RC, Carstens GE, Gutiérrez-Bañuelos H, McReynolds JL, Slay LJ, et al. Effects of oral nitroethane administration on enteric methane emissions and ruminal fermentation in cattle. *Anim Feed Sci Technol* (2011) **166-167**:275-81. doi: 10.1016/j.anifeedsci.2011.04.017.

18. van Zijderveld SM, Gerrits WJJ, Dijkstra J, Newbold JR, Hulshof RBA, Perdok HB. Persistency of methane mitigation by dietary nitrate supplementation in dairy cows. *J Dairy Sci* (2011) **94**:4028-38. doi: 10.3168/jds.2011-4236.

19. Mitsumori M, Shinkai T, Takenaka A, Enishi O, Higuchi K, Kobayashi Y, et al. Responses in digestion, rumen fermentation and microbial populations to inhibition of methane formation by a halogenated methane analogue. *Brit J Nutr* (2012) **108**:482-91. doi: 10.1017/S0007114511005794.

20. Abecia L, Toral PG, Martín-García AI, Martínez G, Tomkins NW, Molina-Alcaide E, et al. Effect of bromochloromethane on methane emission, rumen fermentation pattern, milk yield, and fatty acid profile in lactating dairy goats. *J Dairy Sci* (2012) **95**:2027-36. doi: dx.doi.org/ 10.3168/jds.2011-4831.

21. Hulshof RB, Berndt A, Gerrits WJ, Dijkstra J, van Zijderveld SM, Newbold JR, et al. Dietary nitrate supplementation reduces methane emission in beef cattle fed sugarcane-based diets. *J Anim Sci* (2012) **90**:2317-23. doi: 10.2527/jas.2011-4209.

22. Li L, Davis J, Nolan J, Hegarty R. An initial investigation on rumen fermentation pattern and methane emission of sheep offered diets containing urea or nitrate as the nitrogen source. *Anim Prod Sci* (2012) **52**:653-8. doi: 10.1071/AN11254.

23. Shinkai T, Enishi O, Mitsumori M, Higuchi K, Kobayashi Y, Takenaka A, et al. Mitigation of methane production from cattle by feeding cashew nut shell liquid. *J Dairy Sci* (2012) **95**:5308-16. doi: 10.3168/jds.2012-5554.

24. Martinez-Fernandez G, Abecia L, Martin-Garcia AI, Ramos-Morales E, Hervas G, Molina-Alcaide E, et al. In vitro-in vivo study on the effects of plant compounds on rumen fermentation, microbial abundances and methane emissions in goats. *Animal* (2013) **7**:1925-34. doi: 10.1017/s1751731113001699.

25. El-Zaiat HM, Araujo RC, Soltan YA, Morsy AS, Louvandini H, Pires AV, et al. Encapsulated nitrate and cashew nut shell liquid on blood and rumen constituents, methane emission, and growth performance of lambs. *J Anim Sci* (2014) **92**:2214-24. doi: 10.2527/jas.2013-7084.

26. Haisan J, Sun Y, Guan LL, Beauchemin KA, Iwaasa A, Duval S, et al. The effects of feeding 3-nitrooxypropanol on methane emissions and productivity of Holstein cows in mid lactation. *J Dairy Sci* (2014) **97**:3110-9. doi: 10.3168/jds.2013-7834.

27. Lund P, Dahl R, Yang H, Hellwing A, Cao B, Weisbjerg M. The acute effect of addition of nitrate on in vitro and in vivo methane emission in dairy cows. *Anim Prod Sci* (2014) **54**:1432-5. doi: 10.1071/AN14339.

28. Newbold JR, van Zijderveld SM, Hulshof RB, Fokkink WB, Leng RA, Terencio P, et al. The effect of incremental levels of dietary nitrate on methane emissions in Holstein steers and performance in Nelore bulls. *J Anim Sci* (2014) **92**:5032-40. doi: 10.2527/jas.2014-7677.

29. de Raphélis-Soissan V, Li L, Godwin IR, Barnett MC, Perdok HB, Hegarty RS. Use of nitrate and *Propionibacterium acidipropionici* to reduce methane emissions and increase wool growth of Merino sheep. *Anim Prod Sci* (2014) **54**:1860-6. doi: 10.1071/AN14329.

30. Reynolds CK, Humphries DJ, Kirton P, Kindermann M, Duval S, Steinberg W. Effects of 3-nitrooxypropanol on methane emission, digestion, and energy and nitrogen balance of lactating dairy cows. *J Dairy Sci* (2014) **97**:3777-89. doi: 10.3168/jds.2013-7397.

31. Romero-Perez A, Okine EK, McGinn SM, Guan LL, Oba M, Duval SM, et al. The potential of 3-nitrooxypropanol to lower enteric methane emissions from beef cattle. *J Anim Sci* (2014) **92**:4682-93. doi: 10.2527/jas2014-7573.

32. Martínez-Fernández G, Abecia L, Arco A, Cantalapiedra-Hijar G, Martín-García AI, Molina-Alcaide E, et al. Effects of ethyl-3-nitrooxy propionate and 3-nitrooxypropanol on ruminal fermentation, microbial abundance, and methane emissions in sheep. *J Dairy Sci* (2014) **97**:3790-9. doi: 10.3168/jds.2013-7398.

33. Mitsumori M, Enishi O, Shinkai T, Higuchi K, Kobayashi Y, Takenaka A, et al. Effect of cashew nut shell liquid on metabolic hydrogen flow on bovine rumen fermentation. *Anim Sci J* (2014) **85**:227-32. doi: 10.1111/asj.12133.

34. Mitsumori M, Matsui H, Tajima K, Shinkai T, Takenaka A, Denman SE, et al. Effect of bromochloromethane and fumarate on phylogenetic diversity of the formyltetrahydrofolate synthetase gene in bovine rumen. *Anim Sci J* (2014) **85**:25-31. doi: 10.1111/asj.12072.

35. Hristov AN, Oh J, Giallongo F, Frederick TW, Harper MT, Weeks HL, et al. An inhibitor persistently decreased enteric methane emission from dairy cows with no negative effect on milk production. *Proc Natl Acad Sci USA* (2015) **112**:10663-8. doi: 10.1073/pnas.1504124112.

36. Lee C, Araujo RC, Koenig KM, Beauchemin KA. Effects of encapsulated nitrate on enteric methane production and nitrogen and energy utilization in beef heifers. *J Anim Sci* (2015) **93**:2391-404. doi: 10.2527/jas.2014-8845.

37. Romero-Perez A, Okine EK, McGinn SM, Guan LL, Oba M, Duval SM, et al. Sustained reduction in methane production from long-term addition of 3-nitrooxypropanol to a beef cattle diet. *J Anim Sci* (2015) **93**:1780-91. doi: 10.2527/jas.2014-8726.

38. Veneman JB, Muetzel S, Hart KJ, Faulkner CL, Moorby JM, Perdok HB, et al. Does dietary mitigation of enteric methane production affect rumen function and animal productivity in dairy cows? *PLoS One* (2015) **10**:e0140282. doi: 10.1371/journal.pone.0140282.

39. Lopes JC, de Matos LF, Harper MT, Giallongo F, Oh J, Gruen D, et al. Effect of 3-nitrooxypropanol on methane and hydrogen emissions, methane isotopic signature, and ruminal fermentation in dairy cows. *J Dairy Sci* (2016) **99**:5335-44. doi: 10.3168/jds.2015-10832.

40. Martinez-Fernandez G, Denman SE, Yang C, Cheung J, Mitsumori M, McSweeney CS. Methane inhibition alters the microbial community, hydrogen flow, and fermentation response in the rumen of cattle. *Front Microbiol* (2016) **7**:1122. doi: 10.3389/fmicb.2016.01122.

41. Olijhoek DW, Hellwing AL, Brask M, Weisbjerg MR, Hojberg O, Larsen MK, et al. Effect of dietary nitrate level on enteric methane production, hydrogen emission, rumen fermentation, and nutrient digestibility in dairy cows. *J Dairy Sci* (2016) **99**:6191-205. doi: 10.3168/jds.2015-10691.

42. Vyas D, McGinn SM, Duval SM, Kindermann M, Beauchemin KA. Effects of sustained reduction of enteric methane emissions with dietary supplementation of 3-nitrooxypropanol on growth performance of growing and finishing beef cattle. *J Anim Sci* (2016) **94**:2024-34. doi: 10.2527/jas.2015-0268.

43. Vyas D, McGinn SM, Duval SM, Kindermann MK, Beauchemin KA. Optimal dose of 3-nitrooxypropanol for decreasing enteric methane emissions from beef cattle fed high-forage and high-grain diets. *Anim Prod Sci* (2016). doi: 10.1071/AN15705.

44. Haisan J, Sun Y, Guan LL, Beauchemin KA, Iwaasa A, Duval S, et al. The effects of feeding 3-nitrooxypropanol at two doses on milk production, rumen fermentation, plasma metabolites, nutrient digestibility, and methane emissions in lactating Holstein cows. *Anim Prod Sci* (2017) **57**:282-9. doi: 10.1071/AN15219.

45. Martinez-Fernandez G, Denman SE, Cheung J, McSweeney C. Phloroglucinol degradation in the rumen promotes the capture of excess hydrogen generated from methanogenesis inhibition. *Front Microbiol* (2017). doi: 10.3389/fmicb.2017.01871.

46. van Nevel CJ, Henderickx HK, Demeyer DI, Martin J. Effect of chloral hydrate on methane and propionic acid in the rumen. *Appl Microbiol* (1969) **17**:695-700.

47. Farra PA, Satter LD. Manipulation of the ruminal fermentation. III. Effect of nitrate on ruminal volatile fatty acid production and milk composition. *J Dairy Sci* (1971) **54**:1018-24.

48. Trei JE, Parish RC, Singh YK. Effect of methane inhibitors on rumen metabolism and feedlot performance of sheep. *J Dairy Sci* (1971) **54**:536-40.

49. Trei JE, Scott GC, Parish RC. Influence of methane inhibition on energetic efficiency of lambs. *J Anim Sci* (1972) **34**:510-5.

50. Johnson ED, Wood AS, Stone JB, Moran Jr ET. Some effects of methane inhibition in ruminants (steers). *Can J Anim Sci* (1972) **52**:703-12. doi: 10.4141/cjas72-083.

51. Lanigan GW. Metabolism of pyrrolizidine alkaloids in the ovine rumen. IV. Effects of chloral hydrate and halogenated methanes on rumen methanogenesis and alkaloid metabolism in fistulated sheep. *Aust J Agric Res* (1972) **23**:1085-91.

52. Clapperton JL. The effect of trichloroacetamide, chloroform and linseed oil given into the rumen of sheep on some of the end-products of rumen digestion. *Brit J Nutr* (1974) **32**:155-61.

53. Czerkawski JW, Breckenridge G. New inhibitors of methane production by rumen micro- organisms. Experiments with animals and other practical possibilities. *Brit J Nutr* (1975) **34**:447-57.

54. Horton GM. A note on the effects of monensis and amicloral in steers diets. *Anim Prod* (1980) **30**:441-4.

55. Horton GM. Use of feed additives to reduce ruminal methane production and deaminase activity in steers. *J Anim Sci* (1980) **50**:1160-4.

56. Kung L, Jr., Smith KA, Smagala AM, Endres KM, Bessett CA, Ranjit NK, et al. Effects of 9,10 anthraquinone on ruminal fermentation, total-tract digestion, and blood metabolite concentrations in sheep. *J Anim Sci* (2003) **81**:323-8. doi: /2003.811323x.

57. Anderson RC, Carstens GE, Miller RK, Callaway TR, Schultz CL, Edrington TS, et al. Effect of oral nitroethane and 2-nitropropanol administration on methane-producing activity and volatile fatty acid production in the ovine rumen. *Bioresource Technol* (2006) **97**:2421-6. doi: 10.1016/j.biortech.2005.10.013.

58. Gutierrez-Banuelos H, Anderson RC, Carstens GE, Slay LJ, Ramlachan N, Horrocks SM, et al. Zoonotic bacterial populations, gut fermentation characteristics and methane production in feedlot steers during oral nitroethane treatment and after the feeding of an experimental chlorate product. *Anaerobe* (2007) **13**:21-31. doi: 10.1016/j.anaerobe.2006.11.002.

59. Knight T, Ronimum RS, Dey D, Tootill C, Naylor G, Evans P, et al. Chloroform decreases rumen methanogenesis and methanogen populations without altering rumen function in cattle. *Anim Feed Sci Technol* (2011) **166-167**:101-12. doi: 10.1016/j.anifeedsci.2011.04.059.

60. Klevenhusen F, Duval S, Zeitz JO, Kreuzer M, Soliva CR. Diallyl disulphide and lovastatin: effects on energy and protein utilisation in, as well as methane emission from, sheep. *Arch Anim Nutr* (2011) **65**:255-66. doi: 10.1080/1745039X.2011.588845.

61. Lin M, Guo W, Meng Q, Stevenson DM, Weimer PJ, Schaefer DM. Changes in rumen bacterial community composition in steers in response to dietary nitrate. *Appl Microbiol Biotechnol* (2013) **97**:8719-27. doi: 10.1007/s00253-013-5143-z.

62. Abecia L, Martín-García AI, Martínez G, Newbold CJ, Yañez-Ruiz DR. Nutritional intervention in early life to manipulate rumen microbial colonization and methane output by kid goats postweaning. *J Anim Sci* (2013) **91**:4832-40. doi: 10.2527/jas.2012-6142.

63. Abecia L, Waddams KE, Martinez-Fernandez G, Martin-Garcia AI, Ramos-Morales E, Newbold CJ, et al. An antimethanogenic nutritional intervention in early life of ruminants modifies ruminal colonization by Archaea. *Archaea* (2014) **2014**:841463. doi: 10.1155/2014/841463.

64. Asanuma N, Yokoyama S, Hino T. Effects of nitrate addition to a diet on fermentation and microbial populations in the rumen of goats, with special reference to *Selenomonas ruminantium* having the ability to reduce nitrate and nitrite. *Anim Sci J* (2015) **86**:378-84. doi: 10.1111/asj.12307.

65. Guyader J, Eugene M, Meunier B, Doreau M, Morgavi DP, Silberberg M, et al. Additive methane-mitigating effect between linseed oil and nitrate fed to cattle. *J Anim Sci* (2015) **93**:3564-77. doi: 10.2527/jas.2014-8196.

66. Lee C, Araujo RC, Koenig KM, Beauchemin KA. Effects of encapsulated nitrate on eating behavior, rumen fermentation, and blood profile of beef heifers fed restrictively or ad libitum. *J Anim Sci* (2015) **93**:2405-18. doi: 10.2527/jas.2014-8851.

67. Troy SM, Duthie CA, Hyslop JJ, Roehe R, Ross DW, Wallace RJ, et al. Effectiveness of nitrate addition and increased oil content as methane mitigation strategies for beef cattle fed two contrasting basal diets. *J Anim Sci* (2015) **93**:1815-23. doi: 10.2527/jas.2014-8688.

68. Zhao L, Meng Q, Ren L, Liu W, Zhang X, Huo Y, et al. Effects of nitrate addition on rumen fermentation, bacterial biodiversity and abundance. *Asian-Australas J Anim Sci* (2015) **28**:1433-41. doi: 10.5713/ajas.15.0091.

69. Nguyen SH, Barnett MC, Hegarty RS. Use of dietary nitrate to increase productivity and reduce methane production of defaunated and faunated lambs consuming protein-deficient chaff. *Anim Prod Sci* (2016) **56**:290-7.

70. Guyader J, Doreau M, Morgavi DP, Gerard C, Loncke C, Martin C. Long-term effect of linseed plus nitrate fed to dairy cows on enteric methane emission and nitrate and nitrite residuals in milk. *Animal* (2016) **10**:1173-81. doi: 10.1017/s1751731115002852.

71. Lee C, Araujo RC, Koenig KM, Beauchemin KA. In situ and in vitro evaluations of a slow release form of nitrate for ruminants: Nitrate release rate, rumen nitrate metabolism and the production of methane, hydrogen, and nitrous oxide. *Anim Feed Sci Technol* (2017) **231**:97-106. doi: 10.1016/j.anifeedsci.2017.07.005.
